# Exploring cation distribution in ion-exchanged Al,Ga-containing metal–organic frameworks using ^17^O NMR spectroscopy†

**DOI:** 10.1039/d3cp03071g

**Published:** 2023-07-18

**Authors:** Zachary H. Davis, Russell E. Morris, Sharon E. Ashbrook

**Affiliations:** a School of Chemistry, EaStCHEM and Centre of Magnetic Resonance, University of St Andrews, St Andrews KY16 9ST UK sema@st-andrews.ac.uk rem1@st-andrews.ac.uk

## Abstract

A mixed-metal metal–organic framework, (Al,Ga)-MIL-53, synthesised by post-synthetic ion exchange has been investigated using solid-state nuclear magnetic resonance (NMR) spectroscopy, microscopy and energy dispersive X-ray (EDX) spectroscopy. ^17^O enrichment during the ion-exchange process enables site specific information on the metal distribution to be obtained. Within this work two ion-exchange processes have been explored. In the first approach (exchange of metals in the framework with dissolved metal salts), ^17^O NMR spectroscopy reveals the formation of crystallites with a core–shell structure, where the cation exchange takes place on the surface of these materials forming a shell with a roughly equal ratio of Al^3+^ and Ga^3+^. For the second approach (exchange of metals between two frameworks), no core–shell structure is observed, and instead crystallites containing a majority of Al^3+^ are obtained with lower levels of Ga^3+^. Noticeably, these particles show little variation in the metal cation distribution between crystallites, a result not previously observed for bulk (Al,Ga)-MIL-53 materials. In all cases where ion exchange has taken place NMR spectroscopy reveals a slight preference for clustering of like cations.

## Abstract 2

^17^O isotopic enrichment at different synthetic points provides insight into the cation distribution in mixed-metal (Al,Ga)-MIL-53 using NMR spectroscopy.

## Graphical abstract

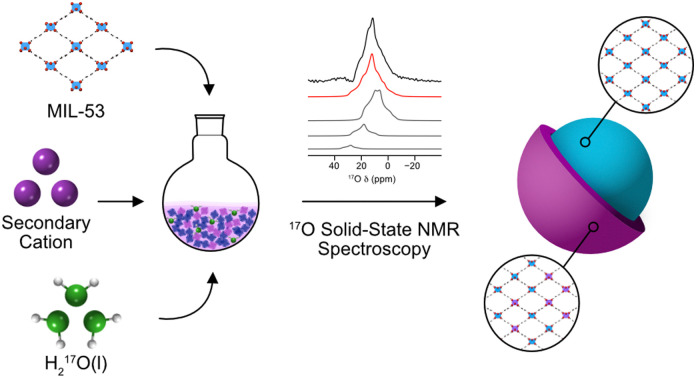

## Introduction

Metal–organic frameworks (MOFs) are a class of microporous materials containing characteristic pores and channels with a size typically less than 20 Å in diameter. These voids can reversibly adsorb small molecules, known as guests. MOFs comprise two key components: nodes, which are either a single metal or cluster of metal cations; and linkers, which are multitopic organic molecules.^1–3^ The interest in MOFs lies in their structural diversity, which can be achieved by varying the metal cation and/or the organic linker, enabling control of the framework structure and its resulting physical and chemical properties. This leads to a wide range of potential applications, with MOFs showing promise in the fields of catalysis,^4–7^ energy materials,^8–10^ gas storage,^11–13^ separation^14–16^ and healthcare.^17,18^

**Fig. 1:**
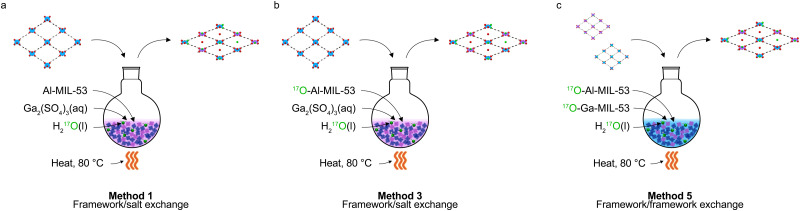
Schematic representing the types of ion exchange processes used in this work. (a) Framework/salt ion exchange (method 1), where a single cation framework (Al-MIL-53) is suspended in a solution containing a secondary cation (Ga^3+^) where the framework is only enriched during the ion exchange step. (b) Framework/salt ion exchange (method 3) where the framework is enriched during the initial MOF synthesis and during the ion exchange step. (c) Framework/framework ion exchange (method 5) where two single cation frameworks with different constituent cations (Al- and Ga-MIL-53) are suspended in a solution, and with the frameworks enriched during the initial MOF synthesis and during the ion exchange step. When Al^3+^ and Ga^3+^ are swapped in (a) and (b) these methods are referred to as 2 and 4.

Interest in mixed-metal^19,20^ and mixed-linker^21^ MOFs, sometimes called multivariate MOFs, lies in the tunability of their properties.^19,22^ For example, additional functionality can be incorporated into a framework through a second ligand type, or by functionalisation of the existing ligand and mixed-metal MOFs have potential for applications within the fields of catalysis,^23–25^ through the introduction of multiple active metal sites.^26^ There is also the possibility of tuning gas adsorption^27,28^ through regulating breathing behaviour and flexibility.^29,30^ Mixed-metal frameworks can be synthesised by one of two possible routes:^19,31^ through a direct one-pot approach,^32,33^ in which two different metal cations are added in the initial framework synthesis or by a post-synthetic ion-exchange process, where either a single cation framework is soaked in a solution containing a second cation (framework/salt ion exchange, as illustrated in Fig. 1a),^34^ or two pre-assembled single cation frameworks with different cations are suspended in a solution (framework/framework ion exchange, as shown in Fig. 1c).^31^ However, analysing these mixed-metal materials is a complex task given the local disorder introduced by adding a second metal cation and requires detailed investigation to fully understand the structure and properties of such materials. Diffraction-based techniques only provide information based on the average structure and are often not sufficient alone to determine the cation distribution in such materials. Less common or more complex techniques are then required to obtain detailed structural information; for example, atom probe tomography can map sequences of metals in multivariate MOFs.^33^ In this paper we show how solid-state NMR spectroscopy can be used to provide a wealth of information on structural details in mixed metal (Al/Ga) MOFs with the MIL-53 structure. As we look to understand the properties of mixed-metal MOFs, ^17^O NMR spectroscopy is shown to be an excellent probe of metal distributions. The results we present here demonstrate that the detailed local structure of post-synthetically modified materials depends greatly on the nature of the ion exchange process allowing for greater understanding of these complex materials.

### MIL-53

A particular group of frameworks that show interesting structural properties are known as breathing MOFs, such as MIL-53 (MIL = Material Institut Lavoisier).^35^ These breathing frameworks exhibit large changes in their pore volumes, up to 40% in the case of Al-MIL-53, depending on external conditions such as temperature, pressure and type of guest molecule present.^35–38^ The breathing behaviour, which determines the size and shape of the pores formed under certain conditions, can also be dependent upon the cation with which the framework is constructed.^32,37,39,40^ MIL-53 is composed of the ditopic linker benzene 1,4-dicarboylate (BDC^2−^) and, when first reported by the Férey group in 2002, the metal node Cr^3+^.^35^ Since then, the synthesis of MIL-53 has been expanded to include Al^3+^, Fe^3+^, In^3+^, Ga^3+^ and Sc^3+^,^36,41–44^ each of which exhibit some variation in their breathing behaviour.^39^ Prior work by Bignami *et al.* and Rice *et al.* explored the effect of the cation ratio in mixed-metal (Al,Ga)-MIL-53 on the overall breathing behaviour of the framework and how this differs from that seen for the parent (*i.e.*, single metal) frameworks, using ^13^C and ^17^O solid-state NMR spectroscopy.^32,37^ Within MIL-53 the metal cations are octahedrally coordinated to O from four separate BDC^2−^linkers and to two bridging hydroxyl groups, forming the three-dimensional (3D) wine-rack like framework. These hydroxyl groups connect the metal nodes together in rows.^35^

### Solid-state NMR spectroscopy

Solid-state NMR spectroscopy is a powerful technique for understanding structure in disorder materials given its sensitivity to the local, atomic-level environment, without the need for any long-range ordering.^45–47^ Previous studies have shown that ^17^O NMR spectroscopy can provide detailed information on MOFs, such as the pore form adopted by breathing frameworks and the metal distribution in mixed-metal materials.^32,37,48^^17^O, the only NMR active isotope of O, brings its own challenges, given its quadrupolar nature (*I* = 5/2), very low natural abundance (0.037%) and only moderate gyromagnetic ratio (*ν*_0_ = 81.36 MHz at 14.1 T). However, the quadrupolar moment of ^17^O (−2.56 fm^2^) is relatively small and thus spectra can be acquired with moderate magnetic field strengths (*i.e.*, 14.1 T) and, in addition, MQMAS experiments can be used to acquire high-resolution, isotropic NMR spectra. The main hurdle to the acquisition of ^17^O NMR spectra is the low natural abundance of ^17^O and thus isotopic enrichment is typically used to enable NMR spectra to be acquired on a reasonable timescale. However, the high cost of isotopically labelled reagents, for example 90% ^17^O H_2_O(l), at €1900 mL^−1^, requires cost effective and atom efficient enrichment processes such as low-level solvent dry-gel conversion (DGC) syntheses and scaled ion-exchange reactions.^32,37,48,49^

Within this work a series of ^17^O-enriched (Al,Ga)-MIL-53 materials have been prepared by the two post-synthetic ion-exchange processes outlined above: framework/salt (Fig. 1a and b) and framework/framework (Fig. 1c) exchange. When using H_2_^17^O(l) as the solvent for the ion-exchange process, ^17^O enrichment of the resulting mixed-metal framework can be achieved. By controlling when ^17^O enrichment of these materials occurs site-specific information can be obtained for ion-exchanged environments, revealing the metal distribution of these mixed-metal materials, for example, during the ion exchange step only (as shown in Fig. 1a) or during both the initial DGC synthesis and ion-exchange step (as shown in Fig. 1b).

## Methods

### Synthesis

Single cation Al- or Ga-MIL-53 were synthesised using either a DGC or hydrothermal approach as reported previously.^32,36,43^ In DGC reactions, Al(NO_3_)_3_·9H_2_O or Ga(NO_3_)_3_·*n*H_2_O (1.38 mmol, Aldrich) was combined with H_2_BDC (1.81 mmol, Aldrich) in a Teflon cup. The Teflon cup was placed inside a 23 mL Parr Teflon-lined autoclave containing H_2_^17^O(l) (130 μL, 90% ^17^O, Cortecnet) before sealing and heating to 220 °C for 72 h. For hydrothermal syntheses, Al(NO_3_)_3_·9H_2_O or Ga(NO_3_)_3_·*n*H_2_O (1.38 mmol, Aldrich) was combined with H_2_BDC (1.81 mmol, Aldrich). The solid reagents were mixed directly with H_2_O(l) (5 mL) in a 23 mL Parr Teflon-lined autoclave. The autoclave was sealed and heated under the same conditions used for DGC. The resulting as-made materials were calcined by heating to 260 °C under vacuum (10^−4^ Torr) for 72 h. Samples were then sealed in an argon atmosphere to prevent hydration. Ion-exchange reactions were carried out by combining either (i) calcined Al-MIL-53 (0.32 mmol) and calcined Ga-MIL-53 (0.32 mmol), (ii) calcined Al-MIL-53 (0.48 mmol) and Ga_2_(SO_4_)_3_ (0.24 mmol, Aldrich); or (iii) calcined Ga-MIL-53 (0.48 mmol) and Al_2_(SO_4_)_3_ (0.24 mmol, Aldrich) in H_2_^17^O (1 mL, 20% ^17^O, Cortecnet) and heating to 80 °C for 5, 10 or 15 days. The resulting samples were washed with minimal amounts of H_2_O(l) and re-calcined using the procedure described above at 250 °C. Table S1 in the ESI† provides a summary of the five ion-exchange methods used in this work. For all synthetic procedures the metal nitrate salts were used in their hydrated forms. Successful preparation of the MIL-53 framework was confirmed using PXRD as described in previous work.^37^ Unless otherwise stated all MIL-53 materials are studied in their calcined forms.

### Solid-state NMR spectroscopy

Solid-state NMR spectra were acquired using either a Bruker Avance III spectrometer equipped with a 14.1 T or 20.0 T wide-bore magnet or a Bruker NEO spectrometer equipped with a 23.5 T narrow-bore magnet. Samples were packed in 3.2 mm ZrO_2_ rotors and magic angle spinning (MAS) NMR spectra were acquired at spinning speeds of 12.5 kHz (^1^H and ^13^C) and 20 kHz (^17^O) using conventional HX probes. Spectra were acquired at Larmor frequencies of 600.13 MHz, 150.87 MHz and 81.34 MHz for ^1^H, ^13^C and ^17^O, respectively, at 14.1 T. High-field ^17^O spectra were acquired at 115.3 MHz and 135.6 MHz at 20.0 T and 23.5 T, respectively. Spectra are referenced to Si(CH_3_)_4_ for ^1^H and ^13^C, using a secondary reference of l-alanine (*δ*(NH̲_3_) = 8.5 ppm, *δ*(C̲H_3_) = 20.5 ppm) and H_2_O̲(l) for ^17^O at room temperature. ^13^C MAS NMR spectra were acquired using cross polarisation (CP)^50^ from ^1^H with a 5 ms contact pulse, ramped for ^1^H (90 kHz), with TPPM-15 ^1^H decoupling^51^ (90 kHz) during acquisition. ^1^H MAS NMR spectra were acquired using a rotor-synchronised spin echo pulse sequence to avoid baseline distortions, with a radiofrequency (rf) field strength of 100 kHz. For ^17^O, spectra were acquired using either a short (0.5 μs or π/14) flip angle single pulse experiment or a spin echo, with a rf nutation rate of ∼70 kHz. ^17^O multiple-quantum MAS^52^ (MQMAS) experiments were carried out using a triple-quantum z-filtered^53^ (0 → ±3 → 0 → 1) pulse sequence, with sign discrimination achieved using States. Rf nutation rates were ∼70 kHz (first two pulses) and ∼12 kHz (CT selective final pulse). Spectra are shown after a shearing transformation to enable projection of the isotropic spectrum directly onto *δ*_1_ and are referenced using the convention in ref. 54.

### Powder X-ray diffraction

Powder X-ray diffraction (PXRD) patterns were acquired on a STOE STADIP diffractometer (Cu K_α1_), monochromated with a curved Ge(111) crystal in transmission Debye–Scherrer mode. PXRD patterns were acquired for as-made Al- and Ga-MIL-53 synthesised by both DGC and hydrothermal methods, packed into 0.5 mm capillary tubes.

### Electron microscopy, energy dispersive X-ray spectroscopy and focused Ion beam experiments

Scanning electron microscopy (SEM) and EDX spectroscopy measurements were performed using a conventional Jeol JSM-IT200 scanning electron microscope. For all SEM and EDX analysis a working distance of 10 mm and acceleration voltage of 20 kV was used. EDX spectra were acquired on several batches of calcined, hydrated MOFs for each composition, with a minimum of 14 crystallites analysed in each instance. Scanning transmission electron microscopy (STEM) and focused ion beam (FIB) experiments were carried out using a Scios dualbeam STEM/FIB instrument. (Al,Ga)-MIL-53 synthesised by framework/salt ion exchange were suspended in epoxy resin. A slice of the epoxy resin/MOF composites were placed in the STEM instrument where a Ga^+^ ion beam was used to cut away the surface of the epoxy resin to reveal a cross sections of the (Al,Ga)-MIL-53 crystallites ahead of STEM and EDX spectroscopy experiments.

## Results and discussion

**Fig. 2:**
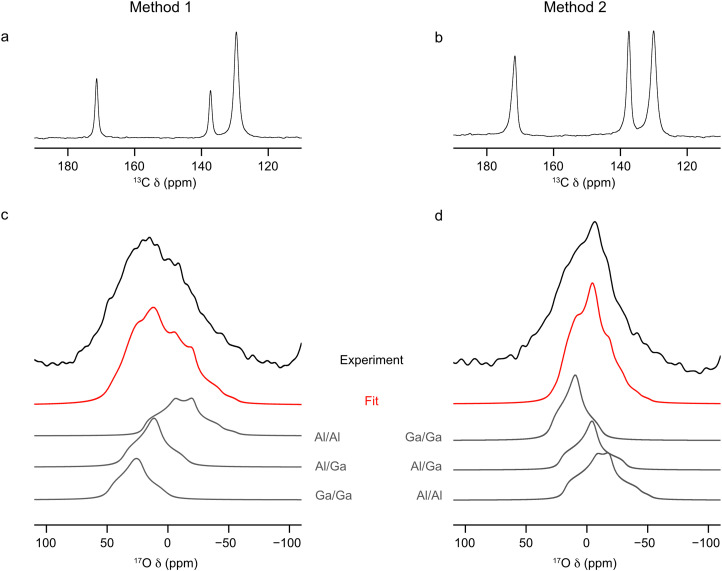
(a and b) ^13^C CP MAS (12.5 kHz, 14.1 T) and (c and d) ^17^O MAS (20 kHz, 14.1 T) NMR spectra (hydroxyl region only and acquired with a short flip angle) of calcined (Al,Ga)-MIL-53 synthesised using (a and c) method 1 and (b and d) method 2, framework/salt ion exchange over a 5 day period, where ^17^O enrichment occurs during the ion-exchange step only. ^17^O MAS NMR spectra acquired by averaging 151,522 transients with a recycle delay of 1 s.

To begin, calcined unenriched single cation Al- and Ga-MIL-53 frameworks were prepared using the hydrothermal synthesis method. The purity of these samples was confirmed by PXRD (see ESI,† Fig. S1) before calcination, following which ^13^C and ^1^H solid-state NMR spectra were acquired (see ESI,† Fig. S3) and compared with previously published data.^32,37^ A calcination temperature of 260 °C was used in order to prevent the partial breakdown of Ga-MIL-53 that occurs at higher temperatures,^37^ affording both Al-MIL-53 and Ga-MIL-53 in the open-pore (OP) form.^37^ Two framework/salt ion-exchange processes were used (see Fig. 1a). In the first, Al-MIL-53 was added to a solution of Ga_2_(SO_4_)_3_ in H_2_^17^O(l) (20% ^17^O) chosen so that the Al and Ga in the system were equimolar (method 1), while in the second, the same process was performed using Ga-MIL-53 and Al_2_(SO_4_)_3_ (method 2). These mixtures were heated for 5 days at 80 °C and the resulting products washed with H_2_O(l) to remove any excess sulfate salts. ^13^C CP MAS and ^17^O MAS NMR spectra of these ion-exchanged materials were acquired before (see ESI,† Fig. S5) and after calcination, Fig. 2. Calcination of the materials was conducted at 250 °C at a reduced pressure of 10^−4^ Torr for 72 h. The lineshapes present in the ^13^C CP MAS NMR spectra before calcination (see ESI,† Fig. S5a and b) are broader than expected for (Al,Ga)-MIL-53, indicating disorder. It should be noted that the chemical shift of the carboxyl C resonance (171 ppm) does not match that seen for the two parent hydrated MIL-53 materials (175 ppm).^37^ These spectra more closely resemble those of as-made MIL-53 materials, suggesting both water and some H_2_BDC linker may be present within the pores of the material. This indicates some free linker may be produced during the ion-exchange process and accounts for the small mass loss (<5%) upon subsequent calcination. Based on previous work, the type of pore form adopted by MIL-53 can be determined from the chemical shifts of the resonances arising from the organic linker present in the ^13^C NMR spectra.^37^ Both materials adopt the OP form following calcination, as determined from the ^13^C CP MAS NMR spectra, shown in Fig. 2a and b, indicating a change in the breathing behaviour from that seen for Ga-MIL-53 prior to ion exchange, suggesting incorporation of Al^3+^ into the framework following method 2 has been successful. The lineshapes in the ^13^C CP MAS NMR spectra are sharper following calcination, indicating an increase in order within the materials due to the removal of the guest molecules. The ^17^O MAS NMR spectra of both materials in Fig. 2 show signal in the range of 50 to −50 ppm, the expected region for hydroxyl O within MIL-53. In both ^17^O MAS NMR spectra acquired before calcination, (see ESI,† Fig. S5c and d) there is a small impurity peak present at 31 ppm. Upon calcination this signal in the ^17^O MAS NMR spectra, disappears indicating the impurity is removed, Fig. 2c and d. There is also a loss of signal in the hydroxyl region upon calcination, suggesting not all of the ^17^O present was contained within the framework hydroxyls, with some likely remaining in the form of guest H_2_^17^O. The presence of hydroxyl signal in the ^17^O MAS NMR spectra following calcination indicates ^17^O enrichment of the frameworks has occurred during the ion-exchange process, showing the lability of the hydroxyl bonds within the material. The relatively low signal-to-noise ratio suggests low overall enrichment of the material, with these ^17^O MAS NMR spectra taking 42 hours to acquire. Comparing the signal-to-noise ratio in these ^17^O MAS NMR spectra with those acquired in previous studies for a direct DGC approach (in which ^17^O enrichment levels was determined to be 20%)^32^ it is estimated these materials have an ^17^O enrichment level of ∼2%. By fitting the lineshapes in the ^17^O MAS NMR spectra acquired with a short flip angle, information on relative proportion of exchanged cation sites can be gathered. The ^17^O MAS NMR parameters determined in previous studies^32,37^ for the three possible types of hydroxyl groups, Al–O(H)–Al, Al–O(H)–Ga and Ga–O(H)–Ga have been used to fit the overall MAS NMR lineshapes, giving the results shown in Table 1. The uncertainty in the fit is greater for the material synthesis using method 1 given the overall broader experimental lineshape (which may arise from an increased level of disorder compared to that of method 2). The overall accuracy in these fits is also hindered by the poor signal-to-noise ratio within the NMR spectra due to low levels of ^17^O in the end materials resulting in a higher uncertainty in the overall percentages of each linkage (being 4% and 3% for frameworks synthesised by methods 1 and 2, respectively). These fits show that using both methods 1 and 2 all three types of hydroxyl groups are present, indicating the MIL-53 framework has been successfully ion exchanged with either Ga^3+^ (method 1) or Al^3+^ (method 2). The level of ion exchange taking place can also be determined from the relative intensities of the three hydroxyl lineshapes. Al : Ga ratios of 58 : 42 and 55 : 45 are found for (Al,Ga)-MIL-53 synthesised by methods 1 and 2, respectively. These values correlate well with the equimolar ratios of Al^3+^ and Ga^3+^ present in both ion-exchange processes. The relative ratios of the signals seen for the three hydroxyl groups indicate a slight preference for clustering of like cations within the material. Should the distribution be random, 49% and 50% of hydroxyls would connect to two different cations for method 1 and method 2 respectively, unlike the 32% and 24% observed, as indicated in Table 1. A plot comparing the relative intensities of the three types of signals observed experimentally to that expected for a random cation distribution is shown in the ESI,† Fig. S10. The extent of this preference for clustering of like cations can be compared to materials reported previously by Bignami *et al.* synthesised using a DGC approach and Rice *et al.* by a hydrothermal method.^32,49^ A plot of the percentage difference between the experimentally determined proportion of Al–O(H)–Ga linkages and the proportion that would be expected if the metal cations were randomly distributed in the framework, ESI,† Fig. S11, shows method 2 produces (Al,Ga)-MIL-53 crystallites with the largest preference for like-cation clustering, with only 48% of the expected proportion of Al–O(H)–Ga linkages compared to a material with a truly random distribution of cations. Crystallites obtained from method 1 contain 66% of the expected proportion of Al–O(H)–Ga linkages for a truly random cation distribution. This can be compared to materials prepared by DGC and hydrothermal approaches, of which 56% and 63% of the expected Al–O(H)–Ga linkages for a fully random cation distribution are present, respectively, indicating a cation clustering preference similar to those observed for method 1. It should be noted that this data only applies to sites where ^17^O exchange has occurred (and as discussed later, reflects only the cation distribution in the shell of these (Al,Ga)-MIL-53 materials).

**Table 1:** ^17^O NMR parameters (isotropic chemical shift (*δ*_iso_), magnitude (*C*_Q_) and asymmetry (*η*_Q_) of the quadrupolar coupling tensor) and relative intensities extracted from fitting the ^17^O MAS NMR spectra (acquired with a short flip angle) of calcined (Al,Ga)-MIL-53 synthesised using methods 1 and 2, framework/salt ion exchange over a 5 day period, where ^17^O enrichment occurs during the ion-exchange step only

Hydroxyl environment	Relative intensity (%)	*δ*_iso_ (ppm)	*C*_Q_/MHz	*η*_Q_
Method 1: Al-MIL-53 + Ga_2_(SO_4_)_3_				
Al–O(H)–Al	42(4)	19(4)	5.7(3)	0.6(2)
Al–O(H)–Ga	32(4)	30(4)	4.4(3)	0.9(2)
Ga–O(H)–Ga	26(4)	39(4)	4.1(3)	0.8(2)

Method 2: Ga-MIL-53 + Al_2_(SO_4_)_3_				
Al–O(H)–Al	43(3)	16(3)	5.4(2)	0.7(2)
Al–O(H)–Ga	24(3)	22(3)	4.5(2)	1.0(2)
Ga–O(H)–Ga	33(3)	35(3)	3.9(2)	1.0(2)

**Fig. 3:**
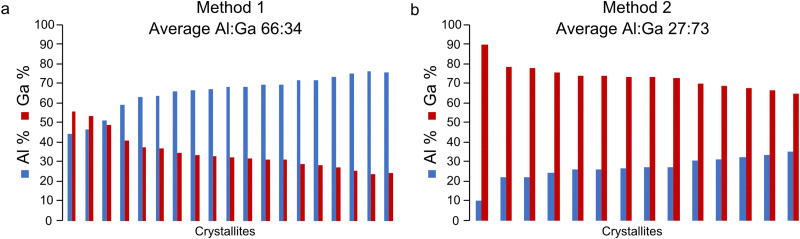
Plots showing the cation composition, determined using EDX spectroscopy, for a range of crystallites of (Al,Ga)-MIL-53 synthesised using (a) method 1 and (b) method 2, framework/salt ion exchange over a 5 day period, where ^17^O enrichment occurs during the ion-exchange step only.

In addition, EDX spectroscopy was performed on both ion-exchanged materials to quantify the Al : Ga ratio for comparison to those determined using ^17^O NMR spectroscopy. EDX spectra were acquired for a range of crystallites (19 for method 1 sample and 14 for method 2 sample) with the relative percentages of Al^3+^ and Ga^3+^ for each crystallite shown in Fig. 3. There is a noticeable spread in the Al : Ga ratio between crystallites for a given material. In the case of method 1, these ratios vary from 45 : 56 to 76 : 24, with an average Al : Ga of 66 : 34 (standard deviation 9.3); and for method 2, these vary between 10 : 90 and 35 : 65, with an average Al : Ga of 27 : 73 (standard deviation 6.2). The variation in cation ratio between crystallites is perhaps not too unexpected given similar ranges were observed for (Al,Ga)-MIL-53 synthesised directly using hydrothermal synthesis and DGC.^37^ Importantly, however, the Al : Ga ratios derived from EDX spectroscopy, which lie in favour of the parent cation, do not match those calculated from fitting ^17^O MAS NMR spectra, which are more representative of the nominal cation amounts used in the synthesis. Therefore, it is evident that these techniques appear to be probing different regions of the (Al,Ga)-MIL-53 crystallites. Given the small size of the (Al,Ga)-MIL-53 particles, which have an average crystallite diameter of 25 μm, the Al : Ga ratio determined by EDX spectroscopy should represent a good average of the cation composition. Hence, the information derived from fitting the ^17^O MAS NMR spectra appears not to be reflective of the entire material, suggesting only O adjacent to exchanged cation sites are enriched during the ion-exchange process. This suggests the formation of crystallites with a core–shell structure, where the core of the material contains only the parent cation and the surface of these materials contains Al^3+^ and Ga^3+^ in a 58 : 42 and 55 : 45 ratio for (Al,Ga)-MIL-53 synthesised by methods 1 and 2, respectively. To investigate this hypothesis further (Al,Ga)-MIL-53 materials were synthesised with the frameworks ^17^O enriched both during the initial MOF synthesis and during the ion-exchange step, to probe all hydroxyl sites present.

**Fig. 4:**
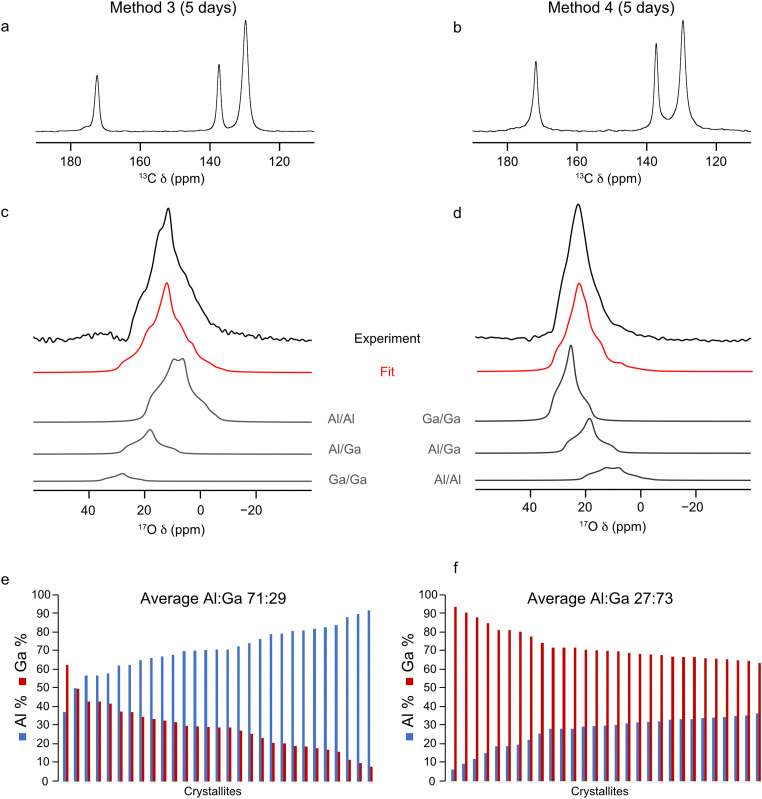
(a) and (b) ^13^C CP MAS (12.5 kHz, 14.1 T), (c) and (d) ^17^O MAS (20 kHz, 23.5 T) NMR spectra (acquired using a short flip angle) and (e) and (f) plots showing the cation composition, determined using EDX spectroscopy, of calcined (Al,Ga)-MIL-53 synthesised using (a), (c), (e) method 3 and (b), (d), (f) method 4, framework/salt ion exchange over a 5 day period, where ^17^O enrichment occurs during both the DGC synthesis and ion-exchange steps. ^17^O MAS NMR spectra acquired by averaging 4096 transients with a recycle delay of 1 s.

^17^O-enriched single cation Al- and Ga-MIL-53 frameworks were prepared using the DGC synthesis method. PXRD patterns were acquired to check the purity and successful synthesis of the as-made materials (see ESI,† Fig. S2) before calcination. Following this, ^13^C, ^1^H and ^17^O MAS NMR spectra were acquired for the calcined frameworks (see ESI,† Fig. S4) which show that ^17^O-Al-MIL-53 adopts the OP form and ^17^O-Ga-MIL-53 the narrow pore (NP) form. However, both materials would be expected to adopt the closed-pore (CP) structure upon exposure to water as part of the ion-exchange process. A sharp resonance is observed at 72 ppm in the ^17^O MAS NMR spectrum of ^17^O-Al-MIL-53, believed to arise from a small aluminium oxide impurity, arising during the framework synthesis. Given the nature of this impurity it is difficult to remove through the calcination procedure as the temperature required would result in the degradation of the MOF itself. Integrating the ^17^O MAS NMR spectrum indicates the impurity is only present as 2(1)% of the total sample. Two subsequent ion-exchange processes were then carried out: the first, in which ^17^O-Al-MIL-53 was mixed into a solution of Ga_2_(SO_4_)_3_ in H_2_^17^O (20% ^17^O) so that the molar amounts of Al and Ga in the system were equal (method 3); and the second, in which the same process was used but for ^17^O-Ga-MIL-53 and Al_2_(SO_4_)_3_ (method 4). Previous studies of ^17^O enrichment of MIL-53 prepared in an identical manner using DGC indicated an ^17^O enrichment level of ∼20%, as measured by mass spectrometry, in the final framework.^32^ Therefore, the solution used for the ion exchange step was also enriched to a similar level in ^17^O. The mixtures were heated for 5 days at 80 °C and the resulting products washed with H_2_O to remove any excess sulfate salts, and ^13^C CP MAS and ^17^O MAS NMR spectra of these materials were acquired after calcination, as shown in Fig. 4a–d. The chemical shifts of the lineshapes present in the ^13^C CP MAS NMR spectra indicate only the OP form is present. The signal-to-noise ratio in the ^17^O MAS NMR spectra is significantly better than that in the spectra acquired for the samples prepared by methods 1 and 2, indicating a higher level of overall ^17^O enrichment within the framework, comparable to the ∼20% achieved in previous studies.^32^ This allows ^17^O MAS NMR spectra of materials synthesised by methods 3 and 4 to be acquired in ∼1 hour.

The NMR parameters used to fit the ^17^O MAS NMR spectra in Fig. 4c and d are shown in Table 2. The increased signal-to-noise ratio in these NMR spectra allow for more accurate fits to be obtained, as reflected in the smaller associated errors (2%) in the hydroxyl group intensities reported in Table 2 compared to those for methods 1 and 2. The intensities of the signals from the three hydroxyl sites indicates an Al : Ga ratio of 86 : 14 and 26 : 74 for materials synthesised using method 3 and 4, respectively. These ratios differ significantly from those determined when ^17^O enrichment occurs during the ion-exchange step only, indicating that not all O sites are enriched as part of this post-synthetic process, leading to site-specific exchange. The results from further EDX spectroscopy experiments, shown in Fig. 4e and f, reveals Al : Ga ratios for these materials which more closely match those determined by ^17^O NMR spectroscopy. (Al,Ga)-MIL-53 prepared by method 3 has an Al : Ga ratio of 71 : 23 (standard deviation 12.3), and by method 4 an Al : Ga ratio of 27 : 73 (standard deviation 8.3) is observed. These ratios lie in favour of the parent cation in both instances, suggesting that, despite the presence of an equimolar amount of Al^3+^ and Ga^3+^ during the ion-exchange process, only a proportion of the secondary cation ends up within the final mixed-metal framework. Once again there is evidence for some clustering of like cations within the materials, with the relative proportion of hydroxyl sites arising from Al–O(H)–Ga groups determined from the NMR spectrum being 20% (method 3) and 29% (method 4), values which are smaller than those expected if there was a completely random distribution of cations, 24% (method 3) and 38% (method 4). This clustering effect would be expected should the crystallites formed during the ion-exchange process constitute a core–shell like structure with the core of the material containing hydroxyl groups connected to the parent cation only (*i.e.*, Al–O(H)–Al in method 3 and Ga–O(H)–Ga in method 4).

**Table 2:** ^17^O NMR parameters and relative intensities extracted from fitting the ^17^O MAS NMR spectra (acquired with a short flip angle) of calcined (Al,Ga)-MIL-53, synthesised using methods 3 and 4, framework/salt ion exchange where ^17^O enrichment occurs during both the DGC synthesis and ion-exchange steps

Hydroxyl environment	Relative intensity (%)	*δ*_iso_ (ppm)	*C*_Q_/MHz	*η*_Q_
Method 3:^17^O-Al-MIL-53 + Ga_2_(SO_4_)_3_				
Al–O(H)–Al	76(2)	19(3)	5.5(2)	0.7(2)
Al–O(H)–Ga	20(2)	28(3)	4.9(2)	1.0(2)
Ga–O(H)–Ga	4(2)^a^	35(3)	3.9(2)	1.0(2)

Method 4:^17^O-Ga-MIL-53 + Al_2_(SO_4_)_3_				
Al–O(H)–Al	11(2)	21(3)	5.4(2)	0.6(2)
Al–O(H)–Ga	29(2)	27(3)	4.9(2)	0.9(2)
Ga–O(H)–Ga	60(2)	32(3)	3.9(2)	1.0(2)

a Note although the fit is slightly better with this component included, the low level of this signal and the presence of an impurity signal resulting from calcination means it is difficult to confirm its presence or accurately determine its intensity.

**Fig. 5:**
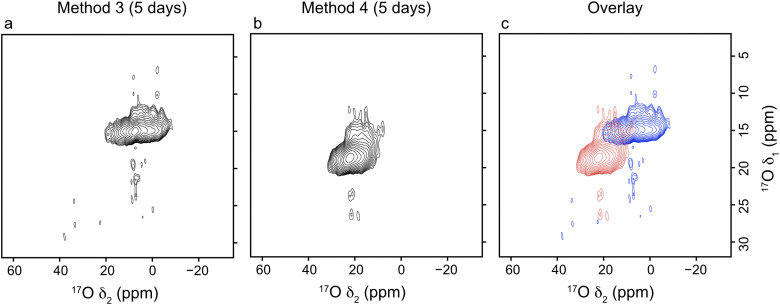
^17^O MQMAS (23.5 T, 20 kHz MAS) of calcined (Al,Ga)-MIL-53 synthesised using (a) method 3 and (b) method 4, framework/salt ion exchange over a 5 day period, where ^17^O enrichment occurs during both the DGC synthesis and ion-exchange steps. (c) Overlay of the two ^17^O MQMAS spectra shown in (a) and (b) with method 3 in red and method 4 in blue. The overlapped region corresponds to signal arising from Al–O(H)–Ga linkages. ^17^O MQMAS NMR spectra acquired by averaging 1024 transients for (a) 140 and (b) 160 t_1_ increments of 12.5 μs with a recycle delay of 0.7 s.

Given the higher level of ^17^O enrichment achieved in these materials, high-resolution ^17^O MQMAS spectra can be acquired on a reasonable timescale, taking ∼28–32 hours per experiment depending on the number of t_1_ increments, as shown in Fig. 5.

**Fig. 6:**
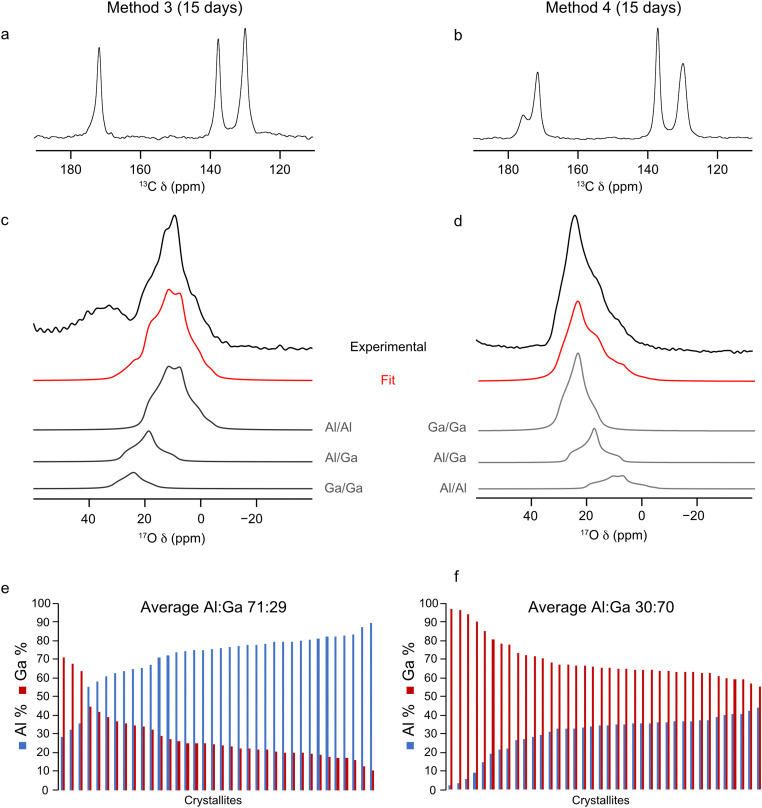
(a) and (b) ^13^C CP MAS (12.5 kHz, 14.1 T), (c) and (d) ^17^O MAS (20 kHz, 23.5 T) NMR spectra (acquired with a short flip angle) and (e) and (f) plots showing the cation composition, determined using EDX spectroscopy, of calcined (Al,Ga)-MIL-53 synthesised using (a), (c), (e) method 3 and (b), (d), (f) method 4, framework/salt ion exchange over a 15 day period, where ^17^O enrichment occurs during both the DGC synthesis and ion-exchange steps. ^17^O NMR spectra acquired by averaging 4096 transients with a recycle delay of 1 s.

Given the range of metal distributions observed between crystallites for these materials (Fig. 4e and f), and the discrepancy observed between the Al : Ga ratio derived from ^17^O NMR and EDX spectroscopy, the ion-exchange process was repeated over a 15 day period to check if additional time would yield different results (*i.e.*, is 5 days sufficient for the ion-exchange process to reach its maximum?). Materials were prepared using method 3 and 4 in order to achieve high levels of ^17^O enrichment to aid with fitting the NMR spectra, with the framework/salt ion exchange mixture being heated for 15 days instead of the typical 5. The ^13^C CP and ^17^O MAS NMR spectra and EDX data acquired for the resulting materials are shown in Fig. 6. Fitting the ^17^O MAS NMR spectra (see ESI,† Table S2, for the NMR parameters used in the fitting) indicate Al : Ga ratios of 89 : 11 and 23 : 77 for the frameworks synthesised by method 3 and 4, respectively, which agrees well with data presented for materials exchanged over 5 days (86 : 14 and 26 : 74, respectively). Likewise, data from EDX spectroscopy reflects relatively similar Al : Ga ratios, which favour the parent cation, of 71 : 29 and 30 : 70 by method 3 and 4 (compared with materials exchanged over 5 days at 71 : 29 and 27 : 73). These comparisons show that the ion-exchange process has reached the limit of secondary cation exchange after 5 days and additional time does not promote further exchange into the MOF framework. Even with the additional reaction time the general profile of distributions of metal ratios between crystallites does not change significantly between 5 and 15 days. Comparing the EDX derived Al : Ga ratios over a range of individual crystallites between samples prepared over 5 and 15 days, as shown in Fig. 4e, f and 6e, f, indicates similar distributions, as evidenced by comparable standard deviations and maximum, minimum and median values (see ESI,† Table S4), confirming the ion-exchange process has reached an end point after 5 days, and that further reaction time does not promote the formation of more homogeneously distributed cations within these materials. Likewise, as seen previously, these materials show the same slight preference for clustering of like cations within the frameworks, as it would be expected for a random cation distribution that 19% and 35% of the hydroxyl groups arise from Al–O(H)–Ga linkages, compared to the values of 13% and 27% determined from the NMR spectra for materials synthesised by method 3 and 4, respectively. High-resolution ^17^O MQMAS NMR spectra of these materials (see ESI,† Fig. S6) can also be acquired, given the higher level of ^17^O enrichment achieved when enriching during both the DGC synthesis and ion-exchange steps. These are comparable with those obtained for (Al,Ga)-MIL-53 synthesised over a 5 day ion exchange period. The third signal present in the region of *δ*_1_ between 14 and 18 ppm, arising due to Al–O(H)–Ga linkages, is more clearly resolved in the ^17^O MQMAS spectrum of (Al,Ga)-MIL-53 synthesised using method 4 at 15 days compared with that in the sample exchanged for only 5 days; however, this does not translate into an increased proportion of Al–O(H)–Ga linkages upon fitting the ^17^O MAS NMR spectrum. It should be noted here the ^13^C CP MAS NMR spectrum of (Al,Ga)-MIL-53 synthesised from ^17^O-Ga-MIL-53 and Al_2_(SO_4_)_3_ (method 4) in Fig. 6b shows the presence of two different pore forms of the MOF, as indicated by the splitting of the carboxyl carbon signal into two resonances at 176 and 172 ppm corresponding to the CP and OP forms, respectively. This is the result of the material partially hydrating after being unpacked from the NMR rotor and subsequently repacked between the acquisition of the ^17^O and ^13^C CP MAS NMR spectra. (Al,Ga)-MIL-53 synthesised from ^17^O-Al-MIL-53 and Ga_2_(SO_4_)_3_ (method 3) was treated in the same manner; however, it does not appear to have hydrated to any observable extent.

**Fig. 7:**
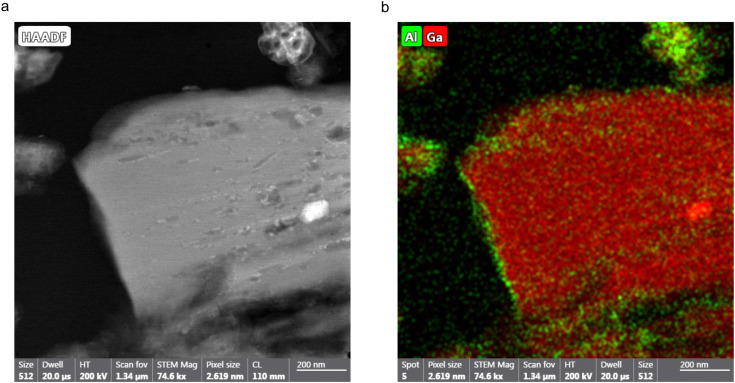
(a) STEM image of a cross section of a (Al,Ga)-MIL-53/epoxy resin composite synthesised using method 4. A Ga^+^ FIB was used to prepare the cross section prior to STEM experiments. (b) An elemental map, acquired using EDX spectroscopy, of the same cross-sectional area showing the presence of Al, in green, and Ga, in red.

Comparing the ^17^O NMR spectroscopy results obtained when ^17^O enrichment occurs during the ion-exchange process only (methods 1 and 2) with those when enrichment occurs during both the DGC synthesis and ion-exchange steps (methods 3 and 4) more detailed information on the overall metal distribution within these frameworks can be obtained. During methods 1 and 2, as discussed earlier, only hydroxyl sites adjacent exchanged cation sites appear to be ^17^O enriched. ^17^O NMR spectra indicate materials synthesised using these methods have Al : Ga ratios of 58 : 42 (method 1) and 55 : 45 (method 2), roughly reflecting the 50 : 50 molar ratio of Al^3+^ and Ga^3+^ present in the reaction. However, methods 3 and 4 show that, when there is uniform distribution of ^17^O within the framework the actual Al : Ga ratios for these materials are different, with much more of the parent cation: 86 : 14 (method 3); and 26 : 74 (method 4). Therefore, it can be seen that the ion-exchange process does not occur throughout the crystallites but is localised to the surface of these material. To confirm this hypothesis STEM and FIB experiments were undertaken to analyse a cross section of one of the MOF crystallites. Fig. 7a shows a STEM image of a cross section of the MOF prepared by method 4, embedded in epoxy resin. Elemental mapping using EDX spectroscopy of these cross sections was undertaken to identify areas of the particle which contained Al and Ga. As shown in Fig. 7b, for (Al,Ga)-MIL-53 synthesised using method 4, Al, in green, is localised to the surface of the crystallite, whereas Ga, in red, is shown to be present throughout the particle, confirming that the ion-exchange process only takes place on the surface. Care needs to be taken when analysing this data given the use of Ga^+^ in the FIB, which will deposit small amounts of Ga across the sample surface. However, this will not affect the distribution of Al seen by EDX spectroscopy, and therefore, we can still conclude that the framework/salt ion-exchange process discussed here occurs primarily at the surface layers, forming a shell containing both Al^3+^ and Ga^3+^ cations. The ESI,† Fig. S13, contains STEM and elemental mapping images for (Al,Ga)-MIL-53 synthesised using method 3. It is challenging to determine the shell size from the STEM and EDX images as the orientation of the particle cross section relative to the electron beam is unknown and the image does not contain the whole crystallite. However, for the image in Fig. 7b, the shell width can be estimated to be ∼45 nm, as measured over the shortest surface distance (*i.e.*, along the left-hand side of the particle). It should be noted that this is a measurement for only one crystallite and therefore not reflective of the bulk material. Additionally, as it can be seen within the STEM image, there is variation in crystallite sizes, complicating any attempt to quantity the bulk material using this methodology. However, it is possible to achieve a bulk estimate of the shell size using the ^17^O NMR spectra reported earlier. This approach assumes that the Al : Ga ratio determined for the sample synthesised using method 1 is localised to ion-exchanged sites (*i.e.*, the shell), but for samples synthesised using method 3 the ratio is representative of the whole framework (*i.e.*, the shell and core). For Al-MIL-53 exchanged with Ga^3+^ (*i.e.*, samples prepared using methods 1 and 3), comparing the relative Al : Ga ratios determined from the ^17^O NMR spectra it can be calculated that the average depth of the shell is 3.1 μm, 12.8% of the crystallite radius. This calculation assumes the average crystallite is spherical in shape with a radius of 24.3 μm (determined from SEM measurements of a sample of 30 crystallites), shown in Table S3 (ESI†). For Ga-MIL-53 exchanged with Al^3+^ (as in methods 2 and 4), this calculation determines the shell to be 3.3 μm, for an average crystallite with radius 17.1 μm, which equates to 19.3% of the particle radius. In both cases the average shell size is comparable, with the only difference being the smaller average Ga-MIL-53 particle size compared with that of Al-MIL-53. It can be theorised that the formation of the CP form upon exposure to water, resulting in a hydrogen bonding network between the guest water molecules and the MIL-53 framework, may restrict access of the secondary cation to the crystallite core, thus limiting the extent of the ion-exchange process to the surface only, resulting in materials with a core–shell structure. It would be interesting therefore to use ^17^O solid-state NMR spectroscopy to investigate non-flexible MOFs to see if the ion-exchange process yields different results. It is not uncommon for post-synthetic exchange reactions of MOFs to produce materials with a core–shell structure, through either metal or ligand exchange processes, although detailed information on the framework composition and distribution of components is not always easily available and sometimes has to be inferred from bulk measurements.^55–58^ There is growing interest in frameworks with core–shell structures, enabling additional functionally within a material which in turn leads to enhanced performance, for example, increasing gas adsorption capacity and the number of accessible active sties.^59,60^ This work shows the utility of ^17^O solid-state NMR spectroscopy to follow such reactions by controlling how, when and where ^17^O enrichment occurs, providing detailed information on the local structure.

**Fig. 8:**
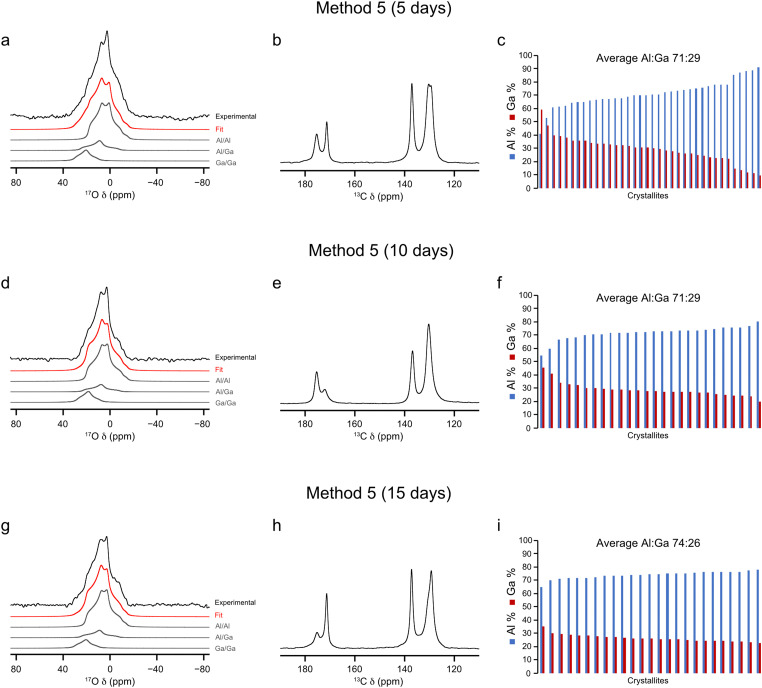
(a), (d) and (g) ^17^O MAS (20 kHz, 20.0 T) acquired with a short flip angle, (b), (e) and h) ^13^C CP MAS (12.5 kHz, 14.1 T) NMR spectra and (c), (f) and (i) plots showing the cation composition, determined using EDX spectroscopy, of calcined (Al,Ga)-MIL-53 synthesised using framework/framework ion exchange over a (a)–(c) 5, (c)–(e) 10 and (g)–(i) 15 day period, where ^17^O enrichment occurs during both the DGC synthesis and ion-exchange steps. ^17^O NMR spectra acquired by averaging 4096 transients with a recycle delay of 1 s.

Exploration of a second type of ion-exchange process, framework/framework, has also been investigated as part of this work. An equimolar amount of ^17^O-enriched Al- and ^17^O-enriched Ga-MIL-53 was suspended in H_2_^17^O(l) (20% ^17^O), heated and allowed to exchange for 5, 10 and 15 day periods (in three individual reactions, method 5). ^17^O MAS and ^13^C CP MAS NMR spectra were acquired of the materials following calcination, as shown in Fig. 8. As observed earlier for one material, there is a splitting of the carboxyl ^13^C signal into two resonances, corresponding to the CP and OP forms, in the three ^13^C CP MAS NMR spectra, indicating partial rehydration between acquisition of the ^17^O MAS and ^13^C CP MAS NMR spectra, as a result of unpacking and repacking the sample. The ^17^O MAS NMR spectra (acquired with a short flip angle) were fitted to extract the relative ratios of the signals from the three types of hydroxyl groups, which are reported in Table 3. These data show a higher average percentage of Al–O(H)–Al linkages in all three samples, starting at 64% after 5 days and ending in 70% after 15 days, showing little overall change with time. The relative average percentages of Al–O(H)–Ga and Ga–O(H)–Ga groups also show little change over the 5, 10 and 15 day periods. These results are interesting as it suggests, despite some crystallites starting with 100% Ga^3+^, that the final crystallites have an average composition containing more Al^3+^cations. As observed for materials synthesised using a framework/salt approach, an amount of free linker is produced during this process. Based on the results discussed earlier in this work, it could be expected that these crystallites might also form a core–shell like structure. From the nature of the ion-exchange method used here (framework/framework) it could be expected that two core–shell arrangements would exist, one with a Al^3+^ core (arising from the initial ^17^O-Al-MIL-53 crystallites) and a second with a Ga^3+^ core (resulting from the ^17^O-Ga-MIL-53 crystallites), each containing a shell with Al^3+^ and Ga^3+^. However, EDX spectroscopy indicates this is not the case. As shown in Fig. 8c, f and i, plots of the three Al : Ga ratios derived from EDX spectroscopy show that all except one of the crystallites analysed contain a majority of Al^3+^. Should crystallites exist which contain a Ga^3+^ core it would be expected that EDX data would show these as having a majority composition of Ga^3+^ over Al^3+^ within the framework, which is not the case. As noted above, free linker is produced during the reaction. It is possible that there is an increased rate of breakdown of Ga-MIL-53 crystallites over that of Al-MIL-53 during the ion-exchange process. Therefore, it would follow that more Ga^3+^ ions are left in solution following the ion-exchange reaction, compared to Al^3+^, resulting in a final framework material which contains more Al^3+^. Previous work shows EDX experiments conducted on mixed-metal (Al,Ga)-MIL-53 provide cation ratios in good agreement with those determined independently using ^17^O NMR, suggesting these are accurate and reliable.^37^ As the time of the ion-exchange process increases, the variation of the Al : Ga ratio between crystallites decreases. This can be quantified in the reduction in the standard deviation over these data sets from 9.9 (5 days) to 5.2 (10 days) to finally 2.8 (15 days). Overall, this evidence would suggest therefore that no core–shell structure is formed during the framework/framework ion-exchange process. No other method of synthesising (Al,Ga)-MIL-53 results in a cation distribution between crystallites that is as uniform as is seen here, including DGC and hydrothermal synthesis methods reported previously by Bignami *et al.* and Rice *et al.* respectively,^32,37^ opening up new potential methods for controlling the metal cation distribution in MOFs. It should be noted that these materials do exhibit a preference once again for clustering of like cations, as is common to all (Al,Ga)-MIL-53 materials prepared here, as evidenced by the relative proportions of the three types of hydroxyl linkages determined by NMR spectroscopy (see ESI,† Fig. S10). The preference for clustering of like cations is greatest for these materials synthesised by method 5 when compared with methods 1 and 2 (as well as DGC and hydrothermal approaches)^32,37^ with crystallites containing 43% (5 days), 31% (10 days) and 40% (15 days) of the expected proportion of Al–O(H)–Ga linkages for a material with truly randomly distributed cations, as shown in the ESI,† Fig. S11. This is perhaps not too unexpected given the ion-exchange process in method 5 starts with two single-metal frameworks.

**Table 3:** ^17^O NMR parameters and relative intensities extracted from fitting the ^17^O MAS NMR spectra (short flip angle) of calcined (Al,Ga)-MIL-53 synthesised using method 5, framework/framework ion exchange, where ^17^O enrichment occurs during the DGC synthesis and ion-exchange steps

Hydroxyl environment	Relative intensity (%)	*δ*_iso_ (ppm)	*C*_Q_/MHz	*η*_Q_
Method 5:^17^O-Al-MIL-53 + ^17^O-Ga-MIL-53, 5 days				
Al–O(H)–Al	64(2)	18(3)	5.4(2)	0.7(1)
Al–O(H)–Ga	17(2)	22(4)	4.9(2)	1.0(2)
Ga–O(H)–Ga	19(2)	31(4)	3.9(3)	1.0(2)

Method 5:^17^O-Al-MIL-53 + ^17^O-Ga-MIL-53, 10 days				
Al–O(H)–Al	68(2)	19(3)	5.4(2)	0.7(1)
Al–O(H)–Ga	12(2)	21(4)	4.9(2)	1.0(2)
Ga–O(H)–Ga	20(2)	34(4)	3.9(2)	1.0(2)

Method 5:^17^O-Al-MIL-53 + ^17^O-Ga-MIL-53, 15 days				
Al–O(H)–Al	70(2)	18(3)	5.4(2)	0.7(1)
Al–O(H)–Ga	14(2)	22(4)	4.9(2)	1.0(2)
Ga–O(H)–Ga	16(2)	31(4)	3.9(2)	1.0(2)

## Conclusions

By conducting ^17^O enrichment during a post-synthetic ion-exchange process to synthesise mixed-metal (Al,Ga)-MIL-53 detailed information on the structure and metal distribution in these complex materials can be obtained. The use of H_2_^17^O as the solvent during the ion-exchange step leads to ^17^O enrichment of the hydroxyl sites within these frameworks, indicating the lability of these groups at 80 °C. It is perhaps not surprising to observe this exchange given the ability for the metal nodes to also interchange between Al^3+^ and Ga^3+^. However, very little ^17^O enrichment of the carboxyl sites is observed using this method, as a result of the stronger C–O bonds present within the linker. The ^17^O enrichment levels achieved are comparable to those obtained in previous studies using a direct DGC approach (∼20%) for methods 3, 4 and 5,^32^ enabling ^17^O NMR spectra to be acquired on a reasonable timescale, with ^17^O MAS and MQMAS NMR experiments taking ∼1 and ∼32 hours respectively, from which information on the relative proportions of the three hydroxyl signals, Al–O(H)–Al, Al–O(H)–Ga and Ga–O(H)–Ga, can be extracted. Materials synthesised by methods 1 and 2 have a significantly lower ^17^O enrichment level, estimated as ∼2%, as seen by the poor signal-to-noise ratio in ^17^O MAS NMR spectra, as enrichment is limited to the shell of these particles. This indicates that the ion-exchange process has successfully produced (Al,Ga)-MIL-53, showing the presence of hydroxyl groups bonded to the secondary cation in all cases.

For framework/salt ion-exchange, when ^17^O enrichment occurs during the ion-exchange step only, the Al : Ga ratio determined from ^17^O MAS NMR experiments disagrees with that from EDX spectroscopy. This suggests that ^17^O enrichment only occurs at exchanged metal cation sites and so is more localised to the surface of the MIL-53 crystallites. ^17^O NMR spectroscopy shows that at the surface the Al : Ga ratio is ∼50 : 50 for exchange methods 1 and 2. When ^17^O enrichment occurs during the initial MOF synthesis (*i.e.*, using DGC and enriched solvent) in addition to the ion-exchange process, information on all hydroxyl sites, not just those actively exchanged during the later step, can be obtained. The enhanced level of ^17^O enrichment observed overall for these materials allows not only ^17^O MAS, but also ^17^O MQMAS NMR spectra to be acquired, in which three distinct resonances can be observed in the hydroxyl region. The Al : Ga ratio determined from ^17^O NMR spectroscopy now matches that from EDX, indicating the formation of MIL-53 crystallites with an overall Al : Ga ratio of 86 : 14 and 26 : 74 using method 3 and 4 respectively, reflecting the parent cation. Extending the ion-exchange process to 15 days (from 5) shows no change in the level of the secondary cation substituted into the MIL-53 framework in both cases, with similar Al : Ga ratios observed irrespective of the ion-exchange time. It is suggested that the adoption of the CP phase by MIL-53 upon exposure to water hinders the accessibility of the secondary cation to metal nodes within the core of the structure, may limit the exchange process to the surface of the crystallites. ^17^O NMR and EDX spectroscopy show the formation of MIL-53 crystallites with a core–shell structure for framework/salt ion-exchange. Within these structures, the core contains only the parent metal cation, while the shell consists of a ∼50 : 50 ratio of Al^3+^ and Ga^3+^. Additionally, ^17^O NMR spectra can also provide information on the average shell size relative to the overall particle radius, determined to be ∼13% and ∼19% for methods 3 and 4, respectively.

For materials synthesised using a framework/framework ion-exchange approach, no core–shell structure is observed. Instead, particles with a majority Al^3+^ content are obtained, despite the equimolar mixture of the frameworks used initially, with only one crystallite analysed by EDX spectroscopy containing over 50% Ga^3+^. Increasing the length of time for the ion-exchange step increases the composition homogeneity between the crystallites, and after 15 days the distribution in the Al : Ga ratio between crystallites is small, ranging from 78 : 22 to 65 : 35. These results show that the framework/framework ion-exchange methodology may be a potential avenue for synthesising mixed-metal MOFs with low variation in cation content between crystallites.

The two ion-exchange methodologies studied here are complicated in nature, and further research needs to be undertaken to better understand the underlying mechanisms taking place. However, this work has illustrated the use of ^17^O NMR spectroscopy as a technique for characterising disordered materials which, in combination with microscopy and EDX spectroscopy, creates a powerful toolkit for structural chemists. By controlling when ^17^O enrichment takes place the ion-exchange process can be followed directly providing detail on the local, atomic scale giving site-specific information on the metal cation distribution, that is that is not usually readily available using other characterisation techniques. Given the increasing interest in more compositionally complex MOFs it is important to characterise how metals are distributed within these materials if we are to understand how the results of different synthesis methods and how the resulting properties of a material vary with composition. This work demonstrates how ^17^O NMR spectroscopy can be used to develop such a better understanding and demonstrates the power the technique has in unravelling the obvious complexities of these fascinating materials.

## Data availability

The research data supporting this publication can be accessed at https://doi/org/10.17630/2fe0818f-9866-4de5-97fb-07bc68692f94.^61^

## Author contributions

Experimental work was carried out by ZHD with the project co-conceived and co-supervised by SEA and REM. The manuscript was written through contributions of all authors. All authors have given approval to the final version of the manuscript.

## Conflicts of interest

There are no conflicts to declare.

## Supplementary Material

CP-025-D3CP03071G-s001
