# Supplementary material for: Exploring cation distribution in ion-exchanged Al,Ga-containing metal–organic frameworks using 17O NMR spectroscopy
Source: Phys Chem Chem Phys. 2023 Jul 18;25(30):20267–80. doi: 10.1039/d3cp03071g (PMC10395001; doi:10.1039/d3cp03071g)
Supplement: CP-025-D3CP03071G-s001 [file CP-025-D3CP03071G-s001.pdf]

Electronic Supplementary Material (ESI) for Physical Chemistry Chemical Physics.
This journal is © the Owner Societies 2023

Supplementary Information: Exploring Cation Distribution in Ion-Exchanged Al,Ga-containing Metal-Organic Frameworks using ^{17}O NMR Spectroscopy

Zachary H. Davis, Russell E. Morris, and Sharon E. Ashbrook

School of Chemistry, EaStCHEM and Centre of Magnetic Resonance, University of St Andrews, St Andrews, KY16 9ST, United Kingdom

S1:	Synthesis Methods	S2
S2:	Powder X-ray Diffraction characterisation of Al- and Ga-MIL-53	S3
S3:	Solid-state NMR characterisation of Al- and Ga-MIL-53	S5
S4:	Solid-state NMR characterisation of (Al,Ga)-MIL-53	S7
S5:	Metal distribution in (Al,Ga)-MIL-53	S12
S6:	SEM, STEM and EDX spectroscopy analysis of (Al,Ga)-MIL-53	S15
S7:	References	S19

S1: Synthesis Methods

Table S1 is provided as a reference guide to the various ion-exchange methods used within this work.

Table S1 – Description of the various ion-exchange methods used in this work, including the parent MOF material, source of secondary cation and ^{17}O enrichment procedures.

Method	Parent Material	Secondary Cation Source	^{17}O Enrichment
1	Al-MIL-53	$Ga_2(SO_4)_3$	Ion-exchange reaction
2	Ga-MIL-53	$Al_2(SO_4)_3$	Ion-exchange reaction
3	^{17}O-Al-MIL-53	$Ga_2(SO_4)_3$	Initial MOF synthesis and ion-exchange reaction
4	^{17}O-Ga-MIL-53	$Al_2(SO_4)_3$	Initial MOF synthesis and ion-exchange reaction
5	^{17}O-Al-MIL-53	^{17}O-Ga-MIL-53	Initial MOF synthesis and ion-exchange reaction

S2: Powder X-ray Diffraction characterisation of Al- and Ga-MIL-53

Figure S1 shows the powder X-ray diffraction (PXRD) patterns acquired for as-made Al- and Ga-MIL-53 synthesised hydrothermally. Figure S2 shows the PXRD patterns acquired for as-made ^{17}O-Al- and ^{17}O-Ga-MIL-53 synthesised and ^{17}O enriched by DGC. Comparison to published PXRD patterns confirms the synthesis of these materials.[S1]

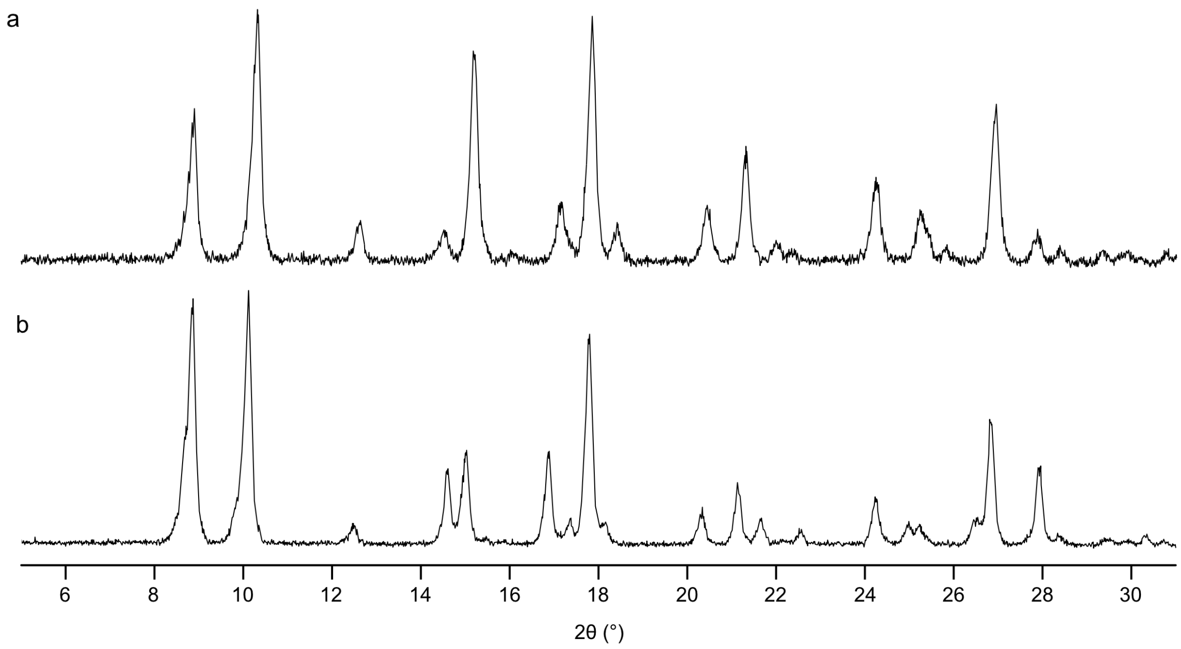

Figure S1 – PXRD patterns for as-made (a) Al- and (b) Ga-MIL-53 synthesised hydrothermally.

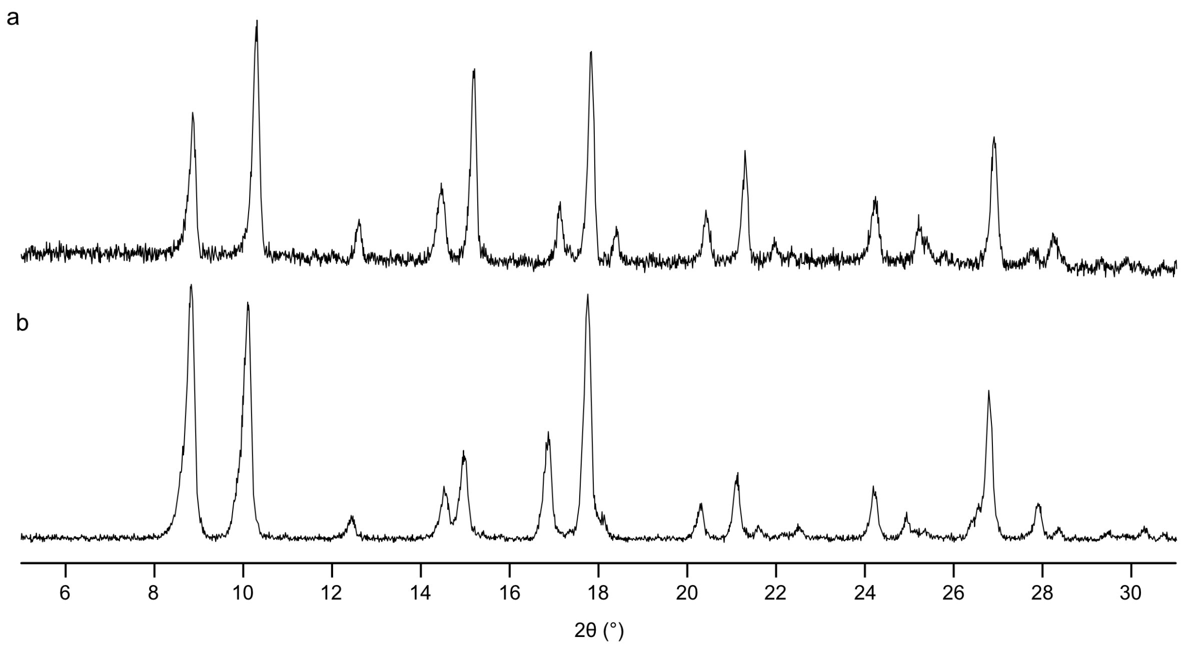

Figure S2 – PXRD patterns for as-made (a) ^{17}O-Al- and (b) ^{17}O-Ga-MIL-53 synthesised and ^{17}O enriched using DGC by DGC.

S3: Solid-state NMR characterisation of Al- and Ga-MIL-53

Figure S3 shows the ^{1}H and ^{13}C CP MAS NMR spectra of calcined Al- and Ga-MIL-53 synthesised hydrothermally and used in the framework/salt ion-exchange processes (methods 1 and 2). ^{1}H MAS NMR spectra confirm there is no water present in the materials, indicating successful calcination. The peaks at 7.8 and 2.3 ppm arise from the hydrogen environments in the linker and hydroxyl groups respectively.[S2] However, there is the presence of a peak at −0.3 ppm, which appears with differing intensities between the two materials, being more prominent in the ^{1}H MAS NMR spectrum of Ga-MIL-53. The ^{13}C CP MAS NMR spectra show the presence of three distinct lineshapes arising from the three unique carbon environments within the BDC linker.[S2,3] Both Al- and Ga-MIL-53 adopt the OP form after calcination (evidenced by the carboxyl carbon peak at 171 ppm).[S1]

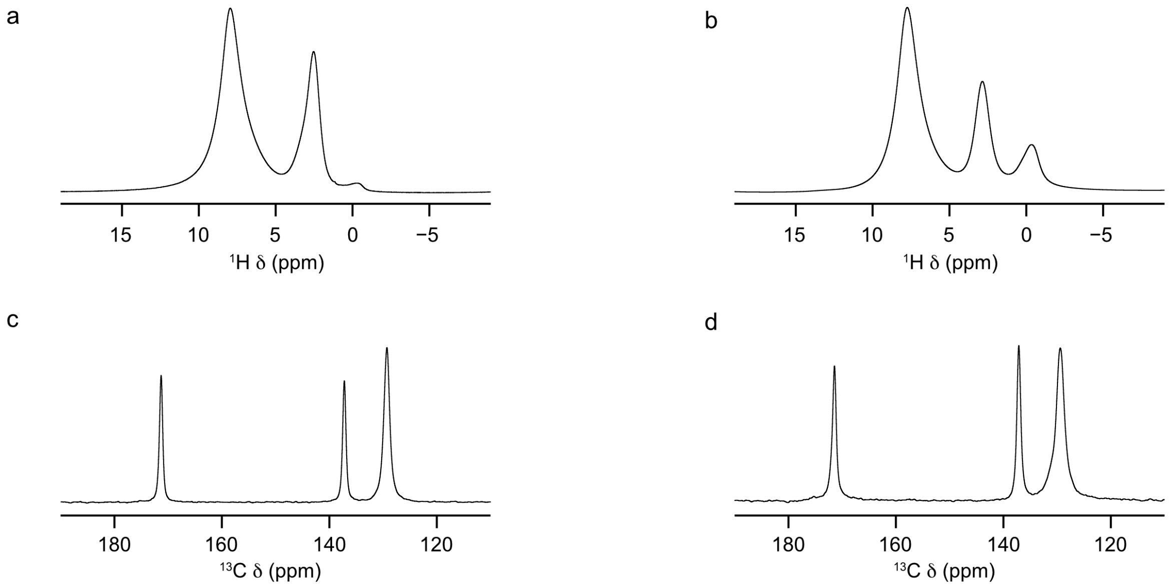

Figure S3 – (a, b) ^{1}H spin-echo MAS and (c, d) ^{13}C CP MAS (12.5 kHz, 14.1 T) NMR spectra of calcined (a, c) Al- and (b, d) Ga-MIL-53 synthesised using a hydrothermal method.

Figure S4 shows the ^{1}H, ^{13}C CP and ^{17}O MAS NMR spectra calcined ^{17}O-Al- and ^{17}O-Ga-MIL-53 synthesised and ^{17}O enriched by DGC and used in both the framework/salt (methods 3 and 4) and framework/framework (method 5) ion-exchange processes. In the case of ^{17}O-Al-MIL-53 the material adopts the OP form (evidenced by the carboxyl carbon peak at 171 ppm) and ^{17}O-Ga-MIL-53 adopts the NP form (evidenced by the carboxyl carbon peak at 175 ppm).[S1] ^{1}H MAS NMR spectra

confirm there is no water present in the materials, indicating successful calcination. The ^{1}H MAS NMR spectrum of Al-MIL-53 also shows the presence of the additional resonance at −0.3 ppm. The ^{17}O MAS NMR spectra of ^{17}O-Al-MIL-53 and ^{17}O-Ga-MIL-53 show two resonances, one in the carboxyl region (~180 ppm) and one in the hydroxyl region (~0 ppm) as expected for the carboxyl oxygen within the linker and the μ_2-hydroxyl group.[3] An additional, sharp, resonance is observed in the ^{17}O MAS NMR spectrum of ^{17}O-Al-MIL-53, Figure S4a, after calcination at 72 ppm, believed to arise from a small aluminium oxide impurity. This data confirms the synthesis of Al- and Ga-MIL-53 for use in the ion-exchange process.

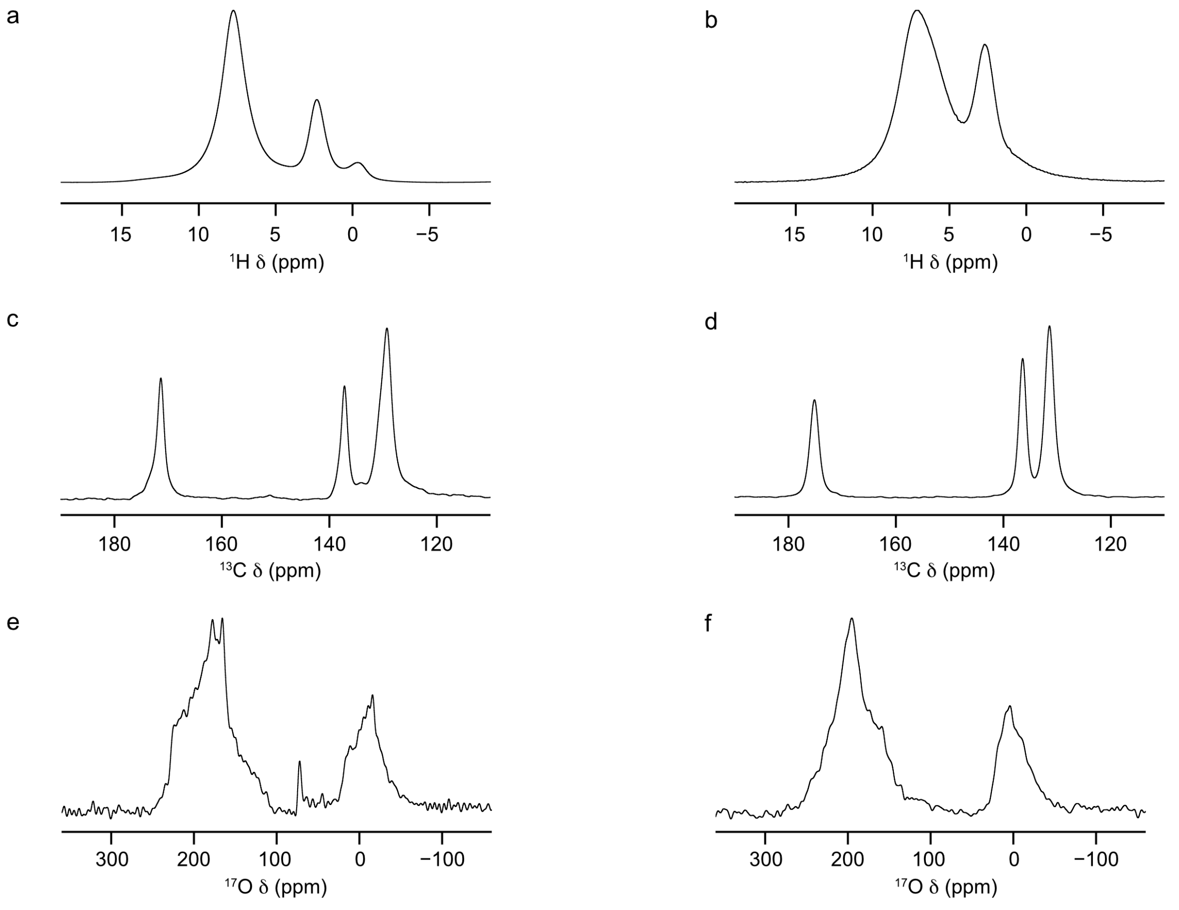

Figure S4 – (a, b) ^{1}H, (c ,d) ^{13}C CP MAS (12.5 kHz, 14.1 T) and (e, f) ^{17}O MAS (20 kHz, 14.1 T) NMR spectra (acquired using a spin echo) of calcined (a, c, e) ^{17}O-Al- and (b, d, f) ^{17}O-Ga-MIL-53 synthesised and ^{17}O enriched using DGC. ^{17}O NMR spectra acquired from averaging 4096 transients with a recycle delay of 1 s.

S4: Solid-state NMR characterisation of (Al,Ga)-MIL-53

Figure S5 shows the ^{13}C CP and ^{17}O MAS NMR spectra of as-made (Al,Ga)-MIL-53 synthesised by the framework/salt approach using methods 1 and 2. Here the broad resonances in the ^{13}C CP MAS NMR spectra are indicative of the as-made form, in which free linker is trapped within the pores of the framework (and can subsequently be removed *via* calcination). Additionally, there is a sharp impurity peak present within the ^{17}O MAS NMR spectra at 31 ppm. It is not known from where this impurity arises; however, it disappears upon calcination.

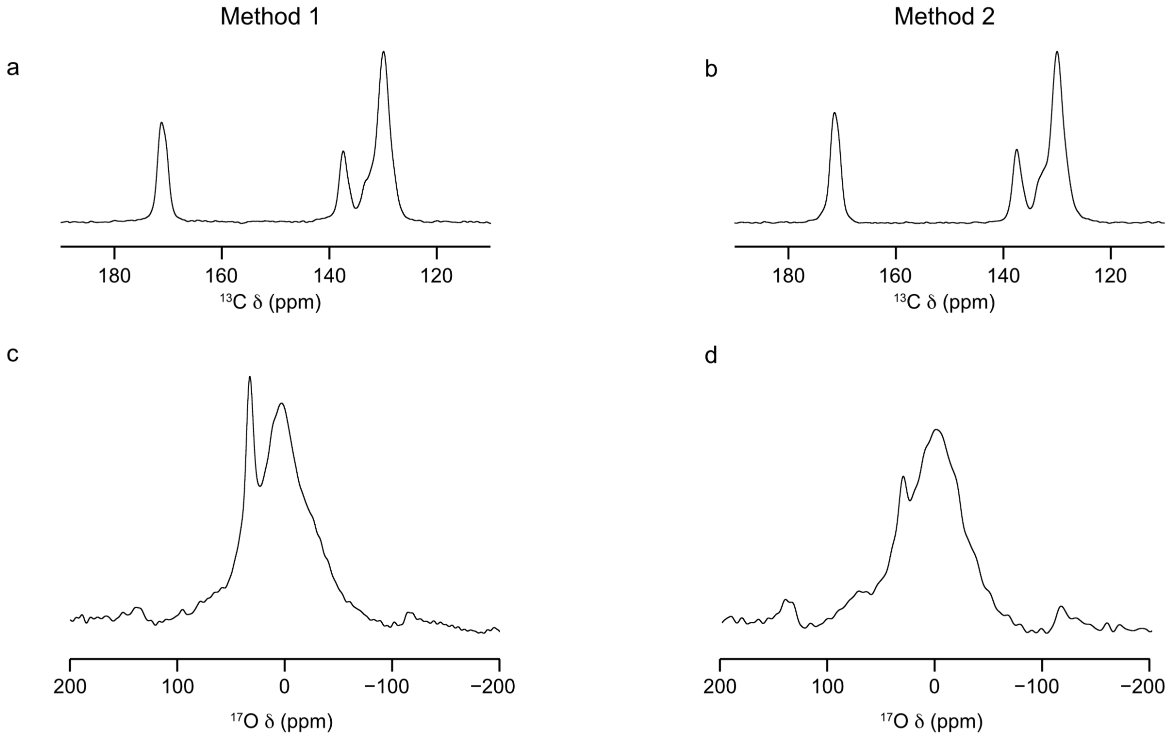

Figure S5 – (a, b) ^{13}C CP MAS (12.5 kHz, 14.1 T) and (c, d) ^{17}O MAS (20 kHz, 14.1 T) NMR spectra (acquired using a spin echo) of (Al,Ga)-MIL-53, pre-calcination, synthesised using (a, c) method 1 and (b, d) method 2, framework/salt ion exchange over a 5 day period, where ^{17}O enrichment occurs during the ion-exchange step only. ^{17}O NMR spectra acquired from averaging 4096 transients with a recycle delay of 1 s.

Figure S6 shows the ^{17}O MQMAS NMR spectra for (Al,Ga)-MIL-53 synthesised using methods 3 and 4 over a 15 day period, and subsequently calcined. These spectra show two main resonances corresponding to Al-O(H)-Al (δ_1 = 14 ppm) and Ga-O(H)-Ga (δ_1 = 18 ppm) linkages in Figures S6a and S6b, respectively. Neither of these resonances appear in the ^{17}O MQMAS NMR spectrum of the opposite compound indicating

relatively little to no presence of the secondary cation in both frameworks. An additional signal can be observed at δ_1 between 14 and 18 ppm, arising from Al-O(H)-Ga linkages, which is most evident upon overlaying the two spectra, as shown in Figure S6c. These spectra are comparable with those acquired for the same materials after 5 days of ion exchange (main text Figure 5), indicating additional reaction time does not promote further ion exchange.

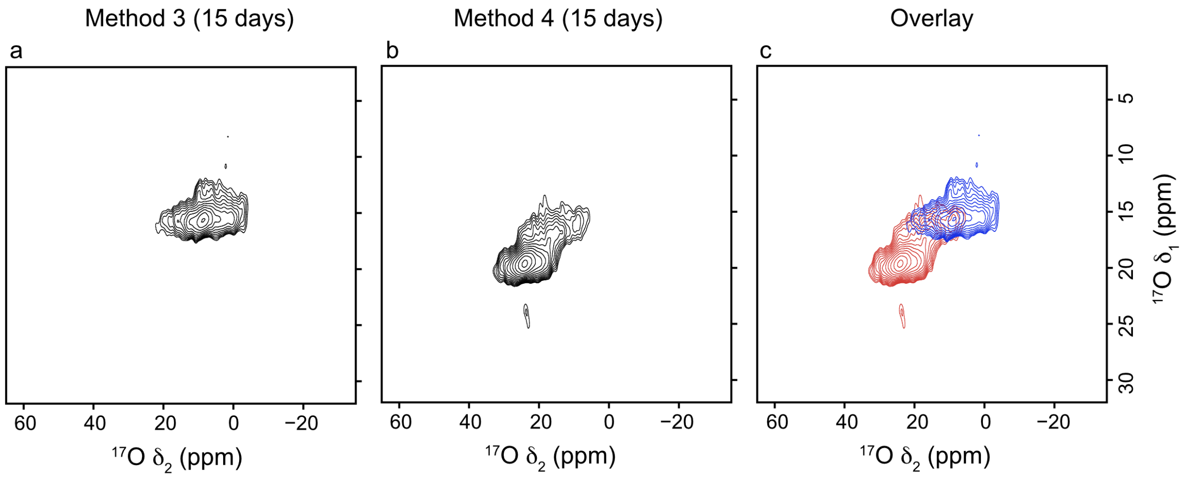

Figure S6 – ^{17}O MQMAS (23.5 T, 20 kHz) NMR spectra of calcined (Al,Ga)-MIL-53 synthesised using (a) method 3 and (b) method 4, framework/salt ion exchange over a 15 day period, where ^{17}O enrichment occurs during both the DGC synthesis and ion exchange steps. (c) Overlay of the two ^{17}O MQMAS spectra shown in (a) and (b) with method 3 in red and method 4 in blue. The overlapped region corresponds to signal arising from Al-O(H)-Ga linkages. ^{17}O MQMAS NMR spectra acquired from averaging 1024 transients for 168 t_1 increments of 12.5 μs with a recycle delay of 0.7 s.

Table S2 – ^{17}O NMR parameters and relative intensities extracted from fitting the ^{17}O NMR spectra (acquired with a short flip angle), shown in Figures 6c,d in the main text, of calcined (Al,Ga)-MIL-53 synthesised using methods 3 and 4, framework/salt ion exchange over a 15 day period, where ^{17}O enrichment occurs during both the initial synthesis and the ion-exchange step.

Hydroxyl environment	Relative intensity (%)	δ_{iso} (ppm)	C_Q / MHz	η_Q
		**Method 3: ^{17}O-Al-MIL-53 + $Ga_2(SO_4)_3$**		
**Al-O(H)-Al**	83(2)	21(3)	5.4(2)	0.6(2)
**Al-O(H)-Ga**	13(2)	28(3)	4.5(2)	1.0(2)
**Ga-O(H)-Ga**	4(2)*	31(3)	3.9(2)	1.0(2)
		**Method 4: ^{17}O-Ga-MIL-53 + $Al_2(SO_4)_3$**		
**Al-O(H)-Al**	9(2)	21(3)	5.4(2)	0.7(2)
**Al-O(H)-Ga**	27(2)	27(3)	4.5(2)	1.0(2)
**Ga-O(H)-Ga**	63(2)	31(3)	3.9(2)	1.0(2)

* Note although the fit is slightly better with this component included, the low level of this signal and the presence of an impurity signal resulting from calcination means it is difficult to confirm its presence or accurately determine its intensity.

Figure S7 shows the complete ^{17}O MAS NMR spectra acquired for calcined (Al,Ga)-MIL-53 synthesised using the two different ion-exchange pathways. When ^{17}O enrichment occurs during the ion-exchange step only, signal is observed in the hydroxyl region (centred at ~ 0 ppm), as seen in Figure S7. There is some signal present in the carboxyl region (centred at ~180 ppm) of the ^{17}O MAS NMR spectrum of (Al,Ga)-MIL-53 synthesised using method 2, indicating possible enrichment of the carboxyl oxygen within the linker; however, this is on a much lower level than that of the hydroxyl group, most likely as a result of the stronger C-O bond, prohibiting the exchange mechanism. When ^{17}O enrichment occurs during both the DGC and ion-exchange steps the carboxyl oxygen signal can clearly be seen at ~180 ppm, Figure S8-S9. ^{17}O enrichment of this site is promoted by the higher temperatures and pressures achieved during the DGC synthesis.

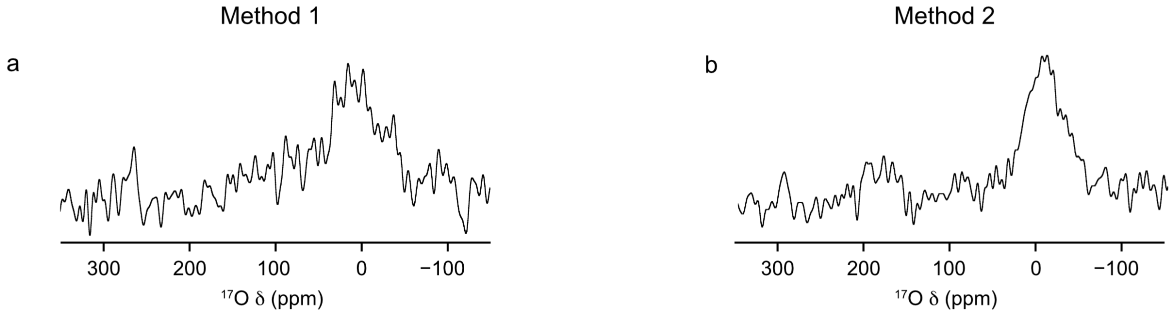

Figure S7 – ^{17}O MAS (20 kHz, 14.1 T) NMR spectra (acquired using a spin echo) of calcined (Al,Ga)-MIL-53 synthesised using (a) method 1 and (b) method 2, framework/salt ion exchange over a 5 day period, where ^{17}O enrichment occurs during the ion-exchange step only. ^{17}O MAS NMR spectra were acquired from averaging 4096 transients with a recycle delay of 1 s.

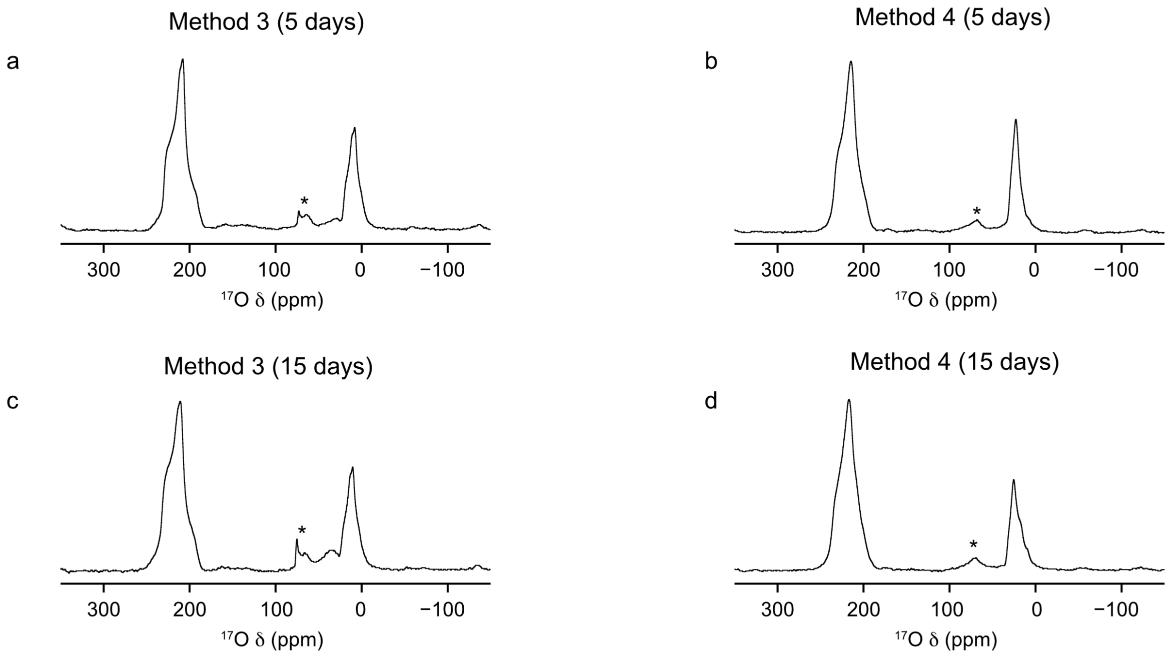

Figure S8 – ^{17}O MAS (20 kHz, 23.5 T) NMR spectra (acquired using a spin echo) of calcined (Al,Ga)-MIL-53 synthesised using (a, c) method 3 and (b, d) method 4, framework/salt ion exchange over a (a, b) 5 and (c, d) 15 day period, where ^{17}O enrichment occurs during both the DGC synthesis and ion-exchange steps. ^{17}O NMR spectra acquired from averaging 4096 transients with a recycle delay of 1 s.

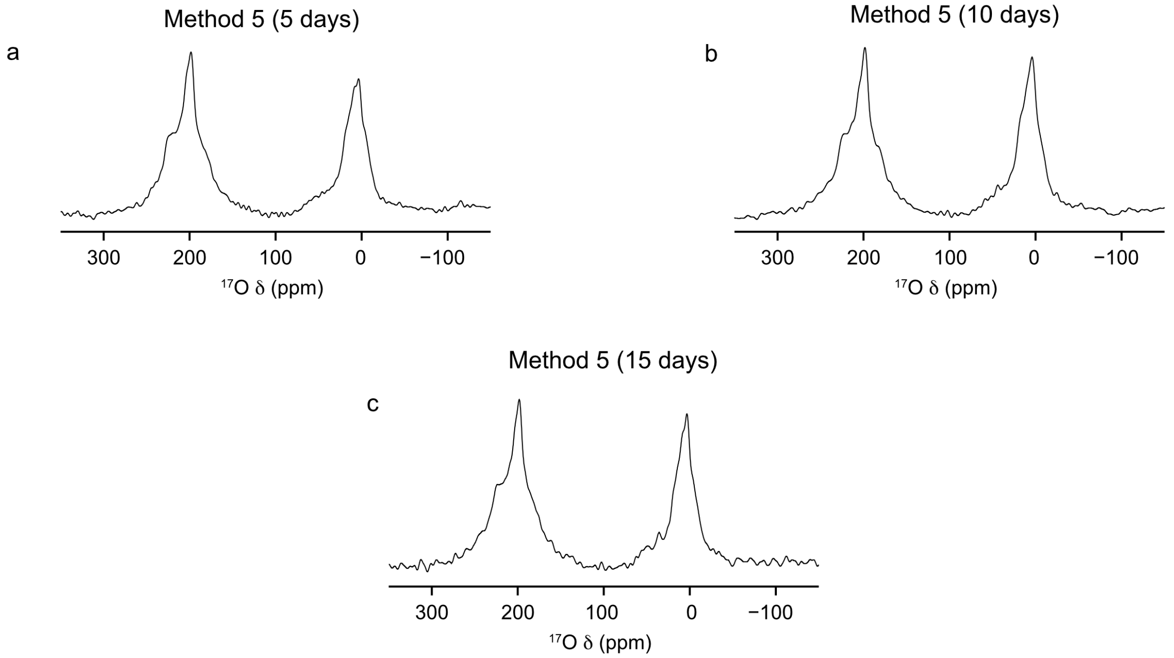

Figure S9 – ^{17}O MAS (20 kHz, 20.0 T) NMR spectra (acquired using a spin echo) of calcined (Al,Ga)-MIL-53 synthesised using method 5, framework/framework ion exchange over a (a) 5, (b) 10 and (c) 15 day period, where ^{17}O enrichment occurs during both the DGC synthesis and ion exchange steps. ^{17}O NMR spectra acquired from averaging 4096 transients with a recycle delay of 1 s.

S5: Metal distribution in (Al,Ga)-MIL-53

Figure S10a shows the relative proportions of the three types of hydroxyl linkages present in all (Al,Ga)-MIL-53 samples, as determined by ^{17}O NMR spectroscopy. When compared to the intensities that would theoretically be expected for a random distribution of cations (for the same composition), Figure S10b, the difference, Figure S10c, indicates in all instances some clustering of like cations occurs within the material, evidenced by a under representation of Al-O(H)-Ga linkages and an over representation of Al-O(H)-Al and Ga-O(H)-Ga linkages.

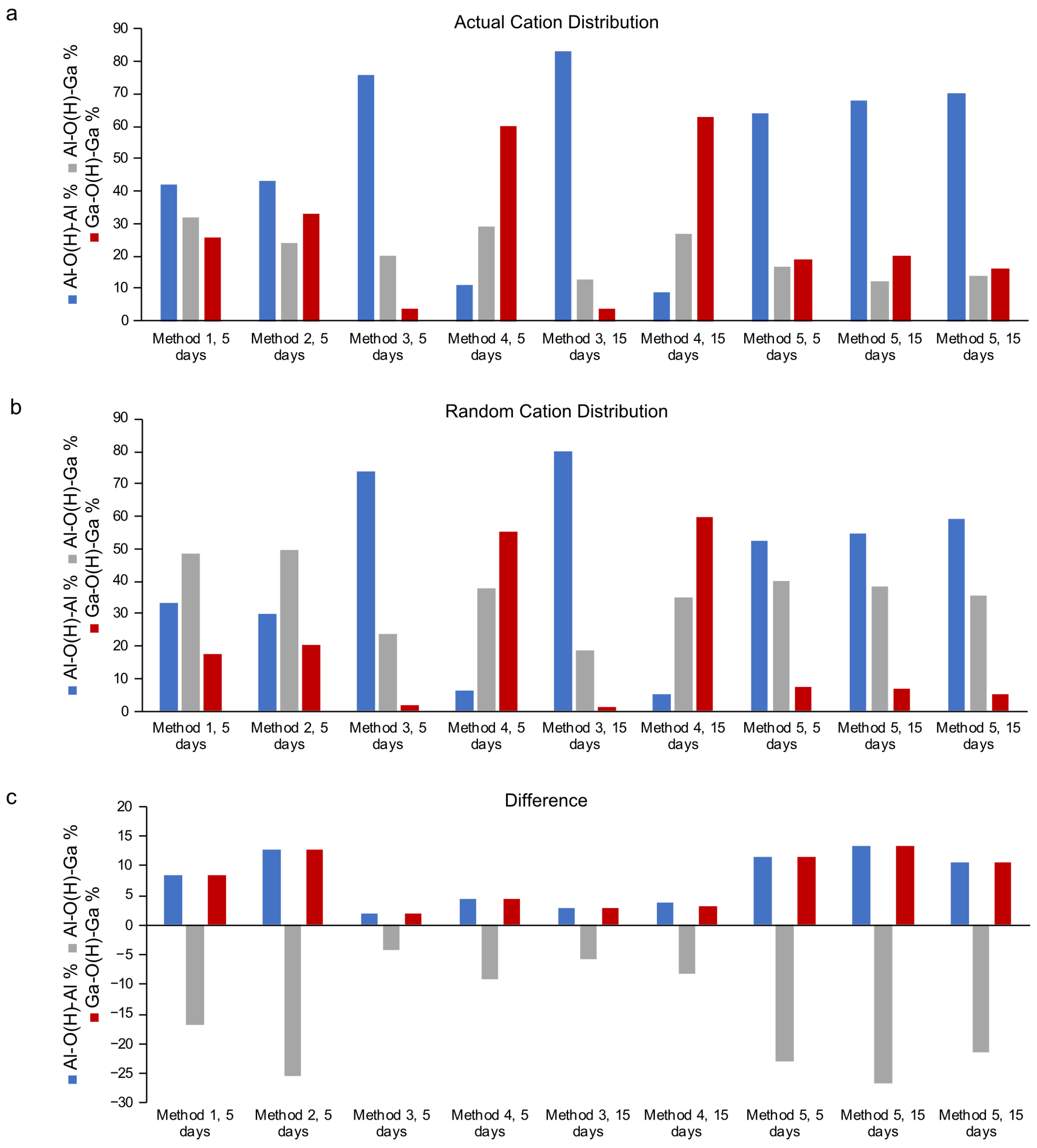

Figure S10 – Plots showing (a) the relative proportions of the three hydroxyl environments, Al-O(H)-Al, Al-O(H)-Ga and Ga-O(H)-Ga, determined by fitting ^{17}O MAS NMR spectra for (Al,Ga)-MIL-53 synthesised *via* a range of ion-exchange processes compared with (b) the predicted proportions of each hydroxyl group expected if the cations were randomly located within the framework. (c) The difference between plots (a) and (b).

Figure S11 shows the percentage difference between the experimentally determined proportion of Al-O(H)-Ga linkages and the proportion that would be expected if the metal cations were randomly distributed in the framework (i.e., $I_{actual} / I_{predicted} \times 100$). Therefore, 100% indicates the cations are truly randomly distributed, with smaller

values suggesting a higher preference for like cation clustering within the framework with 0 representing a fully separated arrangement). Based on this data method 5 produced crystallites with the largest preference for like cation clustering; however, it should be noted that the data presented for methods 1 and 2 only reflects the cation distribution present within the shell of the material. (Al,Ga)-MIL-53 synthesised by method 2 has a shell with higher levels of cation clustering compared to those synthesised using method 1 and direct DGC/hydrothermal approaches, which are more random in nature.

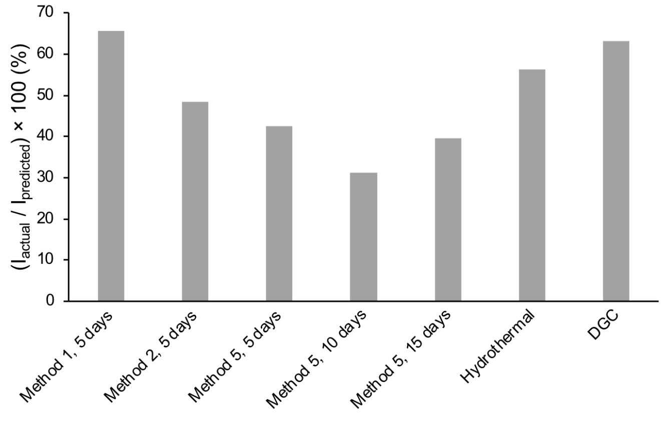

Figure S11 – Plot showing the percentage difference between the experimentally determined proportion of Al-O(H)-Ga linkages and the proportion that would be expected if the metal cations were randomly distributed (Al,Ga)-MIL-53 materials synthesised by methods 1 (crystallite shell only), 2 (crystallite shell only) and 5 as well as direct DGC and hydrothermal approaches. Data for materials synthesised by DGC and hydrothermal methods are obtained from Refs. S3 and S1 respectively.

S6: SEM, STEM and EDX spectroscopy analysis of (Al,Ga)-MIL-53

Table S3 contains a list of crystallite radii for (Al,Ga)-MIL-53 particles synthesised using methods 1 and 2, as measured by SEM, used to calculate the size of the shell of these materials. Histograms of these values shown are shown in Figure S12.

Table S3 – Crystallite radii for a range of (Al,Ga)-MIL-53 particles synthesised using method 1 and 2 and measured by SEM.

Crystallite Number	Radius / μm	
	**Method 1: Al-MIL-53 + $Ga_2(SO_4)_3$**	**Method 2: Ga-MIL-53 + $Al_2(SO_4)_3$**
1	52.0	46.8
2	51.3	40.0
3	31.3	20.7
4	33.3	25.9
5	16.0	26.1
6	15.3	14.3
7	30.0	12.7
8	32.7	19.1
9	13.3	17.0
10	11.3	16.6
11	14.7	16.8
12	31.3	18.9
13	28.0	14.3
14	22.0	12.7
15	30.0	22.5
16	24.7	18.2
17	19.3	9.1
18	18.7	13.4

19	15.3	7.7
20	16.7	10.5
21	39.3	10.7
22	42.7	7.7
23	12.7	9.8
24	10.7	12.3
25	34.7	12.3
26	17.3	8.6
27	8.7	16.6
28	16.0	13.9
29	18.0	19.3
30	22.0	19.8
Average	24.3	17.1

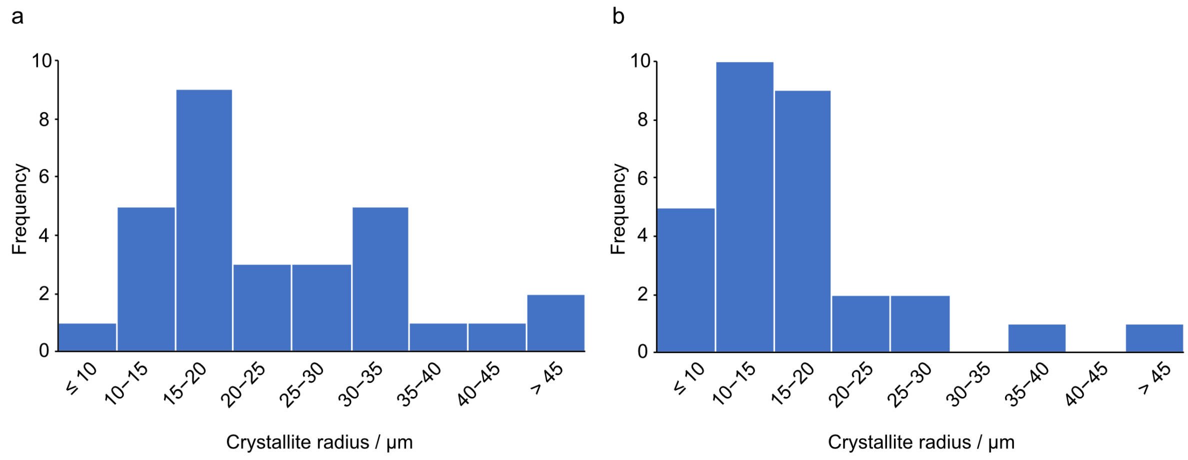

Figure S12 – Histograms showing the distribution of crystallite sizes for (Al,Ga)-MIL-53 synthesised by methods (a) 1 and (b) 2. Data taken from Table S3.

Table S4 – The standard deviation, maximum, minimum and median values of the Al:Ga ratio, acquired using EDX spectroscopy, for (Al,Ga)-MIL-53 synthesised using method 3 and 4 over a 5 and 15 day period.

**Time / days**	**Standard Deviation (Al/Ga%)**	**Maximum**		**Minimum**		**Median**	
		Al%	Ga%	Al%	Ga%	Al%	Ga%
**Method 3:** ^{17}O-**Al-MIL-53 + $Ga_2(SO_4)_3$**							
5	12.3	92.0	62.6	37.4	8.0	70.9	29.1
15	14.4	89.8	71.4	28.6	10.2	76.0	24.0
**Method 4:** ^{17}O-**Ga-MIL-53 + $Al_2(SO_4)_3$**							
5	8.3	36.5	93.8	6.2	63.5	30.0	70.0
15	10.8	44.3	97.4	2.6	55.7	34.6	65.4

Figure S13 shows two cross sections of a (Al,Ga)-MIL-53/epoxy resin composite synthesised *via* method 3 in which ^{17}O-Al-MIL-53 was exchanged with Ga^{3+}. Whilst not as clear as the composite shown in the main paper, Figure 7, for ^{17}O-Ga-MIL-53 exchanged with Al^{3+}, the crystallites can be seen to contain mainly Al, in green, with Ga, in red, present along the surfaces. The use of a Ga^{+} ion beam to mill the surface of the particles to reveal these cross sections leaves a deposit of Ga across the sample, as evidenced by the Ga present outside of the MOF crystallites, and thus care should be taken when drawing conclusions from this data alone.

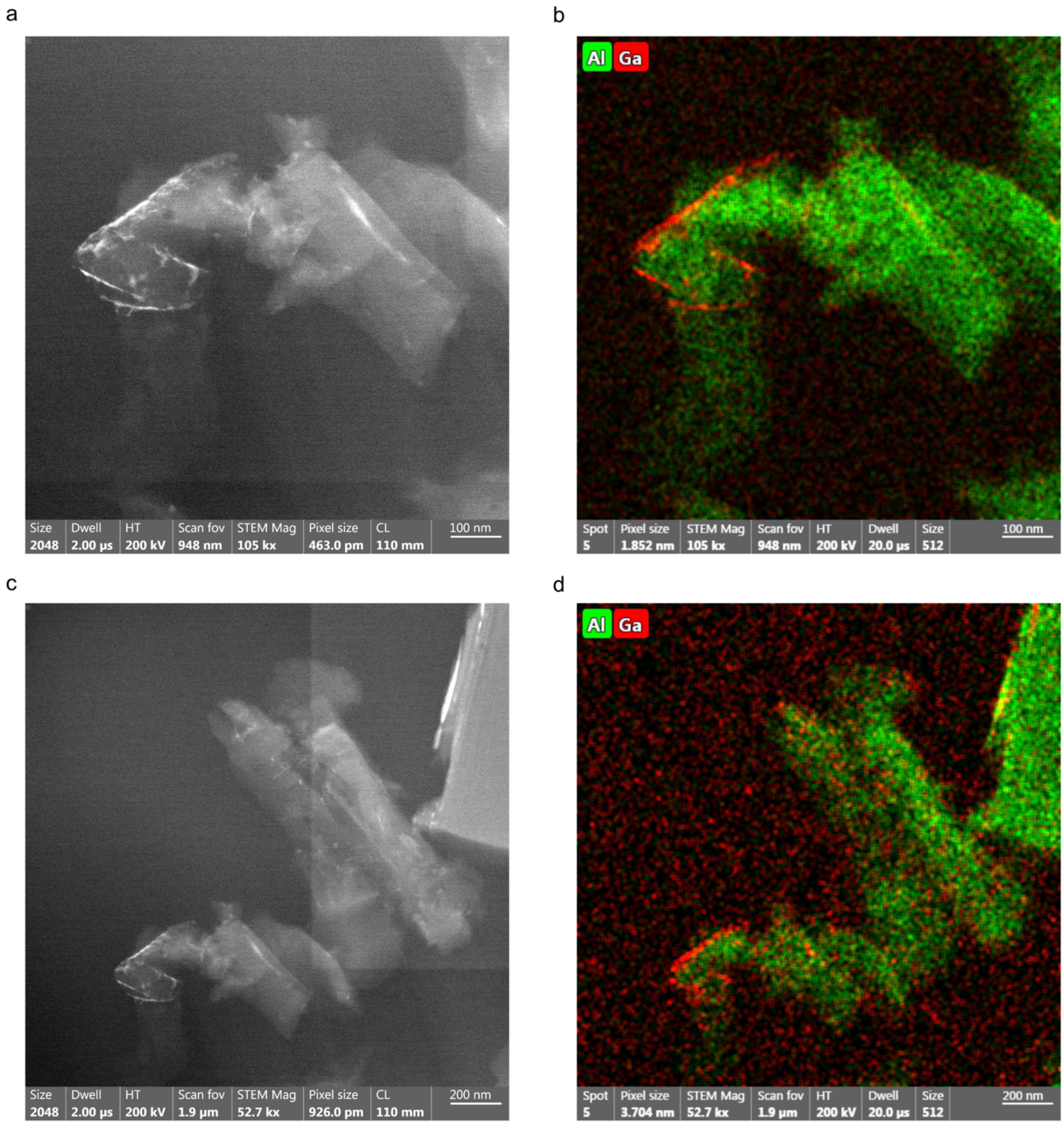

Figure S13 – (a, c) STEM images of a cross section of a (Al,Ga)-MIL-53/epoxy resin composite synthesised using method 3. A Ga^{+} FIB was used to prepare the cross section of sample prior to STEM experiments. (b, d) Elemental maps, acquired using EDX spectroscopy, of the same cross-sectional areas showing the presence of Al, in green, and Ga, in red.

S7: References

1 C. M. Rice, Z. H. Davis, D. McKay, G. P. M. Bignami, R. G. Chitac, D. M. Dawson, R. E. Morris and S. E. Ashbrook, *Phys. Chem. Chem. Phys.*, 2020, **22**, 14514–14526.

2 T. Loiseau, C. Serre, C. Huguenard, G. Fink, F. Taulelle, M. Henry, T. Bataille and G. Férey, *Chem. Eur. J.*, 2004, **10**, 1373–1382.

3 G. P. M. Bignami, Z. H. Davis, D. M. Dawson, S. A. Morris, S. E. Russell, D. McKay, R. E. Parke, D. Iuga, R. E. Morris and S. E. Ashbrook, *Chem. Sci.*, 2018, **9**, 850–859.
